# Auxiliary principle technique and iterative algorithm for a perturbed system of generalized multi-valued mixed quasi-equilibrium-like problems

**DOI:** 10.1186/s13660-017-1324-0

**Published:** 2017-03-04

**Authors:** Mijanur Rahaman, Chin-Tzong Pang, Mohd. Ishtyak, Rais Ahmad

**Affiliations:** 1 Department of Mathematics, Aligarh Muslim University, Aligarh, 202002 India; 2 Department of Information Management, and Innovation Centre for Big Data and Digital Convergence, Yuan Ze University, Chung-Li, 32002 Taiwan

**Keywords:** 35M87, 47H05, 49J40, 65K15, 90C33, quasi-equilibrium-like, perturbed system, algorithm, convergence

## Abstract

In this article, we introduce a perturbed system of generalized mixed quasi-equilibrium-like problems involving multi-valued mappings in Hilbert spaces. To calculate the approximate solutions of the perturbed system of generalized multi-valued mixed quasi-equilibrium-like problems, firstly we develop a perturbed system of auxiliary generalized multi-valued mixed quasi-equilibrium-like problems, and then by using the celebrated Fan-KKM technique, we establish the existence and uniqueness of solutions of the perturbed system of auxiliary generalized multi-valued mixed quasi-equilibrium-like problems. By deploying an auxiliary principle technique and an existence result, we formulate an iterative algorithm for solving the perturbed system of generalized multi-valued mixed quasi-equilibrium-like problems. Lastly, we study the strong convergence analysis of the proposed iterative sequences under monotonicity and some mild conditions. These results are new and generalize some known results in this field.

## Introduction

The theory of variational inequality problem is very fruitful in connection with its applications in economic problems, control and contact problems, optimizations, and many more; see *e.g.*, [1–5]. In 1989, Parida *et al.* [6] introduced and studied the concept of variational-like inequality problem which is a salient generalization of variational inequality problem, and shown its relationship with a mathematical programming problem. One of the most important topics in nonlinear analysis and several applied fields is the so-called equilibrium problem which was introduced by Blum and Oettli [7] in 1994, has extensively studied in different generalized versions in recent past. An important and useful generalization of equilibrium problem is a mixed equilibrium problem which is a combination of an equilibrium problem and a variational inequality problem. For more details related to variational inequalities and equilibrium problems, we refer to [8–15] and the references therein.

There are many illustrious methods, such as projection techniques and their variant forms, which are recommended for solving variational inequalities but cannot be employed in a similar manner to obtain the solution of mixed equilibrium problem involving non-differentiable terms. The auxiliary principle technique which was first introduced by Glowinski *et al.* [16] is beneficial in dodging these drawbacks related to a large number of problems like mixed equilibrium problems, optimization problems, mixed variational-like inequality problems, etc. In 2010, Moudafi [17] studied a class of bilevel monotone equilibrium problems in Hilbert spaces and developed a proximal method with efficient iterative algorithm for solving equilibrium problems. After that, Ding [18] studied a new system of generalized mixed equilibrium problems involving non-monotone multi-valued mappings and non-differentiable mappings in Banach spaces. Very recently, Qiu *et al.* [19] used the auxiliary principle technique to solve a system of generalized multi-valued strongly nonlinear mixed implicit quasi-variational-like inequalities in Hilbert spaces. They constructed a new iterative algorithm and proved the convergence of the proposed iterative method.

Motivated and inspired by the research work mention above, in this article we introduce a new perturbed system of generalized mixed quasi-equilibrium-like problems involving multi-valued mappings in Hilbert spaces. We prove the existence of solutions of the perturbed system of auxiliary generalized multi-valued mixed quasi-equilibrium-like problems by using the Fan-KKM theorem. Then, by employing the auxiliary principle technique and an existence result, we construct an iterative algorithm for solving the perturbed system of generalized multi-valued mixed quasi-equilibrium-like problems. Finally, the strong convergence of iterative sequences generated by the proposed algorithm is proved. The results in this article generalize, extend, and unify some recent results in the literature.

## Preliminaries and formulation of problem

Throughout this article, we assume that $I=\{1,2\}$ is an index set. For each $i\in I$, let $H_{i}$ be a Hilbert space endowed with inner product $\langle\cdot,\cdot\rangle$ and norm $\|\cdot\|$, *d* be the metric induced by the norm $\|\cdot\|$, and let $K_{i}$ be a nonempty, closed, and convex subset of $H_{i}$, $\operatorname{CB}(H_{i})$ be the family of all nonempty, closed, and bounded subsets of $H_{i}$, and for a finite subset *K*, $\operatorname{Co}(K)$ denotes the convex hull of *K*. Let $\mathcal{D}(\cdot,\cdot)$ be the Hausdorff metric on $\operatorname{CB}(H_{i})$ defined by

$$\mathcal{D}(P_{i},Q_{i})=\max \Bigl\{ \sup _{x_{i}\in P_{i}}d(x_{i},Q_{i}), \sup _{y_{i}\in Q_{i}}d(P_{i},y_{i}) \Bigr\} ,\quad\forall P_{i},Q_{i}\in \operatorname{CB}(H_{i}), $$

where $d(x_{i},Q_{i})=\inf_{y_{i}\in Q_{i}}d(x_{i},y_{i})$ and $d(P_{i},y_{i})=\inf_{x_{i}\in P_{i}}d(x_{i},y_{i})$.

For each $i\in I$, let $N_{i}:H_{i}\times H_{i}\longrightarrow\mathbb{R}$ be a real-valued mapping, $M_{i}:H_{i}\times H_{i}\longrightarrow H_{i}$ be a single-valued mapping, $A_{i},T_{i},S_{i}:K_{i}\longrightarrow \operatorname{CB}(H_{i})$ and $B_{i}:K_{1}\times K_{2}\longrightarrow \operatorname{CB}(H_{i})$ be the multi-valued mappings, $\eta _{i}:K_{i}\times K_{i}\longrightarrow H_{i}$ be a nonlinear single-valued mapping, and $f_{i}:K_{i}\longrightarrow K_{i}$ be a single-valued mapping. We introduce the following perturbed system of generalized multi-valued mixed quasi-equilibrium-like problems: Find $(x_{1},x_{2})\in K_{1}\times K_{2}$, $u_{i}\in T_{i}(x_{1})$, $v_{i}\in S_{i}(x_{2})$, $w_{i}\in B_{i}(x_{1},x_{2})$, and $z_{i}\in A_{i}(x_{i})$ such that

1 $$ \textstyle\begin{cases} N_{1}(z_{1},\eta_{1}(f_{1}(y_{1}),f_{1}(x_{1})))+\langle M_{1}(u_{1},v_{1})+w_{1},\eta _{1}(f_{1}(y_{1}),f_{1}(x_{1}))\rangle\\ \quad{}+ \phi_{1}(x_{1},y_{1})-\phi_{1}(x_{1},x_{1})+\alpha_{1}\|f_{1}(y_{1})-f_{1}(x_{1})\|^{2}\geq 0, & \forall y_{1}\in K_{1},\\ N_{2}(z_{2},\eta_{2}(f_{2}(y_{2}),f_{2}(x_{2})))+\langle M_{2}(u_{2},v_{2})+w_{2},\eta _{2}(f_{2}(y_{2}),f_{2}(x_{2}))\rangle\\ \quad{}+\phi_{2}(x_{2},y_{2})-\phi_{2}(x_{2},x_{2})+\alpha_{2}\|f_{2}(y_{2})-f_{2}(x_{2})\|^{2}\geq 0, & \forall y_{2}\in K_{2}, \end{cases} $$

where $\alpha_{i}$ is a real constant and $\phi_{i}:K_{i}\times K_{i}\longrightarrow\mathbb{R}$ is a real-valued non-differential mapping with the following properties:

### Assumption (*)

(i) $\phi_{i}(\cdot,\cdot)$ is linear in the first argument;
(ii) $\phi_{i}(\cdot,\cdot)$ is convex in the second argument;
(iii) $\phi_{i}(\cdot,\cdot)$ is bounded;
(iv) for any $x_{i},y_{i},z_{i}\in K_{i}$,
  $$\phi_{i}(x_{i},y_{i})-\phi_{i}(x_{i},z_{i}) \leq\phi_{i}(x_{i},y_{i}-z_{i}). $$

### Remark 2.1

Notice that the role of the term $\alpha_{i}\|f_{i}(y_{i})-f_{i}(x_{i})\|^{2}$, for each $i\in I$, in problem (1) is analogous to a choice of perturbation in the system. Since $\alpha_{i}$ is a real constant, the solution set of the system (1) is larger than the solution set of system not involving the term $\alpha_{i}\|f_{i}(y_{i})-f_{i}(x_{i})\|^{2}$. It is also remarked that, combining Assumptions (iii) and (iv), it follows that $\phi(\cdot ,\cdot)$ is continuous in the second argument, which is used in many research works; see *e.g.*, [20–23].

Some special cases of the problem (1) are listed below.

(i) If $N_{1}=N_{2}\equiv0$, $f_{1}=f_{2}=I$, the identity mappings, and $\alpha_{1}=\alpha_{2}=0$, then system (1) reduces to the problem of finding $(x_{1},x_{2})\in K_{1}\times K_{2}$, $u_{i}\in T_{i}(x_{1})$, $v_{i}\in S_{i}(x_{2})$, and $w_{i}\in B_{i}(x_{1},x_{2})$ such that
  2 $$ \textstyle\begin{cases} \langle M_{1}(u_{1},v_{1})+w_{1},\eta_{1}(y_{1},x_{1})\rangle+\phi_{1}(x_{1},y_{1})-\phi _{1}(x_{1},x_{1})\geq0, & \forall y_{1}\in K_{1},\\ \langle M_{2}(u_{2},v_{2})+w_{2},\eta_{2}(y_{2},x_{2})\rangle+\phi_{2}(x_{2},y_{2})-\phi _{2}(x_{2},x_{2})\geq0, & \forall y_{2}\in K_{2}. \end{cases} $$
  System (2) was considered and studied by Qui *et al.* [19].
(ii) If $A_{i}$ is a single-valued identity mapping, $f_{i}$ is an identity mapping, $\alpha_{1}=\alpha_{2}=0$, $N_{i}(\cdot,\eta_{i}(f_{i}(y_{i}),f_{i}(x_{i})))=N_{i}(\cdot,y_{i})$, and $w_{i}=-w_{i}\in \operatorname{CB}(K_{i})$, then system (1) reduces to the system of generalized mixed equilibrium problems involving generalized mixed variational-like inequalities of finding $(x_{1},x_{2})\in K_{1}\times K_{2}$, $u_{i}\in T_{i}(x_{1})$ and $v_{i}\in S_{i}(x_{2})$ such that
  3 $$ \textstyle\begin{cases} N_{1}(x_{1},y_{1})+\langle M_{1}(u_{1},v_{1})-w_{1},\eta_{1}(y_{1},x_{1})\rangle+\phi _{1}(x_{1},y_{1})-\phi_{1}(x_{1},x_{1})\geq0, \\ \quad \forall y_{1}\in K_{1},\\ N_{2}(x_{2},y_{2})+\langle M_{2}(u_{2},v_{2})-w_{2},\eta_{2}(y_{2},x_{2})\rangle+\phi _{2}(x_{2},y_{2})-\phi_{2}(x_{2},x_{2})\geq0, \\ \quad \forall y_{2}\in K_{2}. \end{cases} $$
  System (3) introduced and studied by Ding [18].
(iii) If for each $i\in I$, $K_{i}=H_{i}$, $B_{i}\equiv0$, $T_{i}(x_{1})=x_{1}$ and $S_{i}(x_{2})=x_{2}$, then system (2) reduces to the following system of mixed variational-like problems introduced and studied by Kazmi and Khan [22]: Find $(x_{1},x_{2})\in H_{1}\times H_{2}$ such that
  4 $$ \textstyle\begin{cases} \langle M_{1}(x_{1},x_{2}),\eta_{1}(y_{1},x_{1})\rangle+\phi_{1}(x_{1},y_{1})-\phi _{1}(x_{1},x_{1})\geq0, & \forall y_{1}\in H_{1},\\ \langle M_{2}(x_{1},x_{2}),\eta_{2}(y_{2},x_{2})\rangle+\phi_{2}(x_{2},y_{2})-\phi _{2}(x_{2},x_{2})\geq0, & \forall y_{2}\in H_{2}. \end{cases} $$
(iv) If for each $i\in I$, $K_{i}=K$, $N_{i}=N$, $B_{i}=\phi_{i}\equiv 0$, $\alpha_{i}=0$, $A_{i}=A$, $T_{i}=T$, $\eta_{i}=\eta$, $f_{i}=f$ and $\langle M_{i}(u_{i},v_{i}),\eta_{i}(f_{i}(y_{i}),f_{i}(x_{i}))\rangle =M_{i}(u_{i},f_{i}(y_{i}))=M(u,f(y))$, then system (1) equivalent to the problem of finding $x\in K$, $z\in A(x)$ and $u\in T(x)$ such that
  5 $$ N \bigl(z,\eta\bigl(f(y),f(x)\bigr) \bigr)+M\bigl(u,f(y)\bigr) \geq0,\quad\forall y\in K, $$
  which is called the generalized multi-valued equilibrium-like problem, introduced and studied by Dadashi and Latif [24].

It should be noted that, for a suitable choice of the operators $M_{i}, N_{i}, T_{i}, S_{i}, \phi_{i}, \eta_{i}, A_{i}, B_{i}$, and $f_{i}$, for each $i\in I$, in the above mentioned problems, it can easily be seen that the problem (1) covers many known system of generalized equilibrium problems and variational-like equilibrium problems.

Now, we give some definitions and results which will be used in the subsequent sections.

### Definition 2.1

Let *H* be a Hilbert space. A mapping $h:H\longrightarrow\mathbb{R}$ is said to be

(i) upper semicontinuous if, the set $\{x\in H:h(x)>\lambda\}$ is a closed set, for every $\lambda\in\mathbb{R}$;
(ii) lower semicontinuous if, the set $\{x\in H:h(x)>\lambda\}$ is an open set, for every $\lambda\in\mathbb{R}$;
(iii) continuous if, it is both lower semicontinuous and upper semicontinuous.

### Remark 2.2

If *h* is lower semicontinuous, upper semicontinuous, and continuous at every point of *H*, respectively, then *h* is lower semicontinuous, upper semicontinuous, and continuous on *H*, respectively.

### Definition 2.2

Let $\eta:K\times K\longrightarrow K$ and $f:K\longrightarrow K$ be the single-valued mappings. Then *η* is said to be

(i) affine in the first argument if
  $$\eta\bigl(\lambda x+(1-\lambda)z,y\bigr)=\lambda\eta(x,y)+(1-\lambda)\eta (z,y),\quad \forall\lambda\in[0,1], x,y,z\in K; $$
(ii) *κ*-Lipschitz continuous with respect to *f* if there exists a constant $\kappa>0$ such that
  $$\bigl\| \eta\bigl(f(x),f(y)\bigr)\bigr\| \leq\kappa\|x-y\|,\quad\forall x,y\in K. $$

### Definition 2.3

Let $N:H\times H\longrightarrow\mathbb{R}$ be a real-valued mapping and $A:K\longrightarrow \operatorname{CB}(H)$ be a multi-valued mapping. Then *N* is said to be

(i) monotone if
  $$N(x,y)+N(y,x)\leq0,\quad\forall x,y\in H; $$
(ii) *ϱ*-*η*-*f*-strongly monotone with respect to *A* if there exists $\varrho>0$ such that, for any $x,y\in K$, $z\in A(x)$, and $z'\in A(y)$,
  $$N\bigl(z,\eta\bigl(f(y),f(x)\bigr)\bigr)+N\bigl(z',\eta \bigl(f(x),f(y)\bigr)\bigr)\leq-\varrho\bigl\| f(y)-f(x)\bigr\| ^{2}. $$

### Definition 2.4

A mapping $g:K\longrightarrow H$ is said to be

(i) *ε*-*η*-relaxed strongly monotone with respect to *f* if there exists $\varepsilon>0$ such that
  $$\bigl\langle g\bigl(f(x)\bigr)-g\bigl(f(y)\bigr),\eta\bigl(f(x),f(y)\bigr)\bigr\rangle \geq-\varepsilon \bigl\| f(x)-f(y)\bigr\| ^{2}; $$
(ii) *σ*-Lipschitz continuous with respect to *f* if there exists a constant $\sigma>0$ such that
  $$\bigl\| g\bigl(f(x)\bigr)-g\bigl(f(y)\bigr)\bigr\| \leq\sigma\|x-y\|; $$
(iii) hemicontinuous with respect to *f* if, for $\lambda\in [0,1]$, the mapping $\lambda\mapsto g(\lambda f(x)+(1-\lambda)f(y))$ is continuous as $\lambda\to0^{+}$, for any $x,y\in K$.

### Definition 2.5

A mapping $f:H\longrightarrow H$ is said to be *β*-expansive if there exists a constant $\beta>0$ such that

$$\bigl\| f(x)-f(y)\bigr\| \geq\beta\|x-y\|. $$

### Definition 2.6

A multi-valued mapping $P:K\longrightarrow2^{K}$ is said to be KKM-mapping if, for each finite subset $\{x_{1},\ldots,x_{n}\}$ of *K*, $\operatorname{Co}\{x_{1},\ldots,x_{n}\}\subseteq\bigcup_{i=1}^{n} P(x_{i})$, where $\operatorname{Co}\{ x_{1},\ldots,x_{n}\}$ denotes the convex hull of $\{x_{1},\ldots,x_{n}\}$.

### Theorem 2.1

Fan-KKM Theorem [25]

*Let* *K* *be a subset of a topological vector space* *X*, *and let* $P:K\longrightarrow2^{K}$ *be a KKM*-*mapping*. *If for each* $x\in K, P(x)$ *is closed and if for at least one point* $x\in K, P(x)$ *is compact*, *then* $\bigcap_{x\in K}P(x)\neq\emptyset$.

### Definition 2.7

The mapping $M:H\times H \longrightarrow H$ is said to be $(\mu,\xi )$-mixed Lipschitz continuous if, there exist constants $\mu,\xi>0$ such that

$$\bigl\| M(x_{1},y_{1})-M(x_{2},y_{2})\bigr\| \leq\mu \|x_{1}-x_{2}\|+\xi\|y_{1}-y_{2}\|. $$

### Definition 2.8

Let $T:H\longrightarrow \operatorname{CB}(H)$ be a multi-valued mapping. Then *T* is said to be *δ*-$\mathcal{D}$-Lipschitz continuous if, there exists a constant $\delta>0$ such that

$$\mathcal{D} \bigl(T(x),T(y) \bigr)\leq\delta\|x-y\|,\quad\forall x,y\in H, $$

where $\mathcal{D}(\cdot,\cdot)$ is the Hausdorff metric on $\operatorname{CB}(H)$.

### Lemma 2.1

[26]

*Let* $(X,d)$ *be a complete metric space and* $T:X\longrightarrow \operatorname{CB}(X)$ *be a multi*-*valued mapping*. *Then*, *for any given* $\epsilon>0$, $x,y\in X$ *and* $u\in T(x)$, *there exists* $v\in T(y)$ *such that*

$$d(u,v)\leq(1+\epsilon)\mathcal{D}\bigl(T(x),T(y)\bigr). $$

## Formulation of the perturbed system and existence result

In this section, firstly we consider the following perturbed system of auxiliary generalized multi-valued mixed quasi-equilibrium-like problems related to the perturbed system of generalized multi-valued mixed quasi-equilibrium-like problems (1), and prove the existence result.

For each $i\in I$ and given $(x_{1},x_{2})\in K_{1}\times K_{2}$, $u_{i}\in T_{i}(x_{1})$, $v_{i}\in S_{i}(x_{2})$, $w_{i}\in B_{i}(x_{1},x_{2})$ and $z_{i}\in A_{i}(x_{i})$, find $(t_{1},t_{2})\in K_{1}\times K_{2}$ such that, for constants $\rho_{1}, \rho_{2}>0$,

6 $$ \textstyle\begin{cases} \rho_{1}N_{1}(z_{1},\eta_{1}(f_{1}(y_{1}),f_{1}(t_{1})))+\langle g_{1}(f_{1}(t_{1}))-g_{1}(f_{1}(x_{1}))+\rho_{1}(M_{1}(u_{1},v_{1})\\ \quad{}+w_{1}),\eta_{1}(f_{1}(y_{1}),f_{1}(t_{1}))\rangle+\rho_{1} \{\phi_{1}(x_{1},y_{1})-\phi_{1}(x_{1},t_{1})+\alpha_{1}\| f_{1}(y_{1})-f_{1}(t_{1})\|^{2} \}\geq0,\\ \quad \forall y_{1}\in K_{1},\\ \rho_{2}N_{2}(z_{2},\eta_{2}(f_{2}(y_{2}),f_{2}(t_{2})))+\langle g_{2}(f_{2}(t_{2}))-g_{2}(f_{2}(x_{2}))+\rho_{2}(M_{2}(u_{2},v_{2})\\ \quad{}+w_{2}),\eta_{2}(f_{2}(y_{2}), f_{2}(t_{2}))\rangle+\rho_{2} \{\phi_{2}(x_{2},y_{2})-\phi_{2}(x_{2},t_{2})\\ \quad{}+\alpha_{2}\| f_{2}(y_{2})-f_{2}(t_{2})\|^{2} \}\geq0, \\ \quad \forall y_{2}\in K_{2}, \end{cases} $$

where $g_{i}:K_{i}\longrightarrow H_{i}$ is not necessarily the linear mapping. Problem (6) is called the perturbed system of auxiliary generalized multi-valued mixed quasi-equilibrium-like problems. Notice that if $t_{i}=x_{i}$ is a solution of the system (6), then $(x_{i},u_{i},v_{i},w_{i},z_{i})$ is the solution of the system (1).

Now, we establish the following existence and uniqueness of solutions of the perturbed system of auxiliary generalized multi-valued mixed quasi-equilibrium-like problems (6).

### Theorem 3.1

*For each* $i\in I$, *let* $K_{i}$ *be a nonempty*, *closed*, *and convex subset of Hilbert space* $H_{i}$, $N_{i}:H_{i}\times H_{i}\longrightarrow\mathbb{R}$ *be a real*-*valued mapping*, $\phi_{i}:K_{i}\times K_{i}\longrightarrow\mathbb{R}$ *is a real*-*valued non*-*differential mapping*, $M_{i}:H_{i}\times H_{i}\longrightarrow H_{i}$ *be a single*-*valued mapping*, $A_{i},T_{i},S_{i}:K_{i}\longrightarrow \operatorname{CB}(H_{i})$ *and* $B_{i}:K_{1}\times K_{2}\longrightarrow \operatorname{CB}(H_{i})$ *be the multi*-*valued mappings*, $\eta _{i}:K_{i}\times K_{i}\longrightarrow H_{i}$ *be a nonlinear single*-*valued mapping*, *and* $f_{i}:K_{i}\longrightarrow K_{i}$ *be a single*-*valued mapping*. *Assume that the following conditions are satisfied*:

(i) $N_{i}(z_{i},\eta_{i}(f_{i}(x_{i}),f_{i}(x_{i})))=0$, *for each* $x_{i}\in K_{i}$ *and* $N_{i}$ *is convex in the second argument*;
(ii) $N_{i}$ *is* $\varrho_{i}$-$\eta_{i}$-$f_{i}$-*strongly monotone with respect to* $A_{i}$ *and upper semicontinuous*;
(iii) $\eta_{i}$ *is affine*, *continuous in the second argument with the condition* $\eta_{i}(x_{i},y_{i})+\eta_{i}(y_{i},x_{i})=0$, *for all* $x_{i},y_{i}\in K_{i}$;
(iv) $g_{i}$ *is* $\varepsilon_{i}$-$\eta_{i}$-*relaxed strongly monotone with respect to* $f_{i}$ *and hemicontinuous with respect to* $f_{i}$;
(v) $f_{i}$ *is* $\beta_{i}$-*expansive and affine*;
(vi) $\phi_{i}$ *satisfies Assumption* (*);
(vii) $\varepsilon_{i}=\alpha_{i}\rho_{i}$ *and* $3\varepsilon_{i}<\rho _{i}\varrho_{i}$;
(viii) *if there exists a nonempty compact subset* $D_{i}$ *of* $H_{i}$ *and* $t_{i}^{0}\in D_{i}\cap K_{i}$ *such that for any* $t_{i}\in K_{i}\setminus D_{i}$, *we have*
  $$\begin{aligned}& \rho_{i} N_{i} \bigl(z_{i}, \eta_{i} \bigl(f_{i} \bigl(t_{i}^{0} \bigr),f_{i}(t_{i}) \bigr) \bigr)+ \bigl\langle g_{i} \bigl(f_{i} \bigl(t_{i}^{0} \bigr) \bigr)-g_{i}\bigl(f_{i}(x_{i})\bigr)+ \rho_{i} \bigl(M_{i}(u_{i},v_{i}) \\& \quad{}+w_{i} \bigr),\eta_{i} \bigl(f_{i} \bigl(t_{i}^{0} \bigr),f_{i}(t_{i}) \bigr) \bigr\rangle +\rho _{i} \bigl\{ \phi_{i} \bigl(x_{i},t_{i}^{0} \bigr)-\phi_{i}(x_{i},t_{i})+ \alpha_{i} \bigl\Vert f_{i} \bigl(t_{i}^{0} \bigr)-f_{i}(t_{i})\bigr\Vert ^{2} \bigr\} < 0, \end{aligned}$$

*for given* $u_{i}\in T_{i}(x_{1})$, $v_{i}\in S_{i}(x_{2})$, $w_{i}\in B_{i}(x_{1},x_{2})$ *and* $z_{i}\in A_{i}(x_{i})$. *Then the perturbed system of auxiliary generalized multi*-*valued mixed quasi*-*equilibrium*-*like problems* (6) *has a unique solution*.

### Proof

For each $i\in I$, $t_{i}\in K_{i}$ and fixed $(x_{1},x_{2})\in K_{1}\times K_{2}$, $u_{i}\in T_{i}(x_{1})$, $v_{i}\in S_{i}(x_{2})$, $w_{i}\in B_{i}(x_{1},x_{2})$ and $z_{i}\in A_{i}(x_{i})$, define the multi-valued mappings $P_{i}, Q_{i}: K_{i}\longrightarrow2^{K_{i}}$ as follows:

$$\begin{aligned}& P_{i}(y_{i})= \bigl\{ t_{i}\in K_{i}: \rho_{i}N_{i}\bigl(z_{i},\eta _{i}\bigl(f_{i}(y_{i}),f_{i}(t_{i}) \bigr)\bigr)+\bigl\langle g_{i}\bigl(f_{i}(t_{i}) \bigr)-g_{i}\bigl(f_{i}(x_{i})\bigr) \\& \phantom{P_{i}(y_{i})=} {}+\rho_{i}\bigl(M_{i}(u_{i},v_{i})+w_{i} \bigr),\eta_{i}\bigl(f_{i}(y_{i}),f_{i}(t_{i}) \bigr)\bigr\rangle +\rho_{i} \bigl\{ \phi_{i}(x_{i},y_{i})- \phi_{i}(x_{i},t_{i})\\& \phantom{P_{i}(y_{i})=} {}+ \alpha_{i} \bigl\| f_{i}(y_{i})-f_{i}(t_{i}) \bigr\| ^{2} \bigr\} \geq0 \bigr\} , \\& Q_{i}(y_{i})= \bigl\{ t_{i}\in K_{i}: \rho_{i}N_{i}\bigl(z_{i},\eta _{i}\bigl(f_{i}(y_{i}),f_{i}(t_{i}) \bigr)\bigr)+\bigl\langle g_{i}\bigl(f_{i}(y_{i}) \bigr)-g_{i}\bigl(f_{i}(x_{i})\bigr) \\& \phantom{Q_{i}(y_{i})=}{}+\rho_{i}\bigl(M_{i}(u_{i},v_{i})+w_{i} \bigr),\eta_{i}\bigl(f_{i}(y_{i}),f_{i}(t_{i}) \bigr)\bigr\rangle +\rho_{i} \bigl\{ \phi_{i}(x_{i},y_{i})- \phi_{i}(x_{i},t_{i}) \bigr\} \geq0 \bigr\} . \end{aligned}$$

In order to reach the conclusion of Theorem 3.1, we show that all the assumptions of Fan-KKM Theorem 2.1 are satisfied.

First, we claim that $Q_{i}$ is a KKM-mapping. On the contrary, suppose that $Q_{i}$ is not a KKM-mapping. Then there exist $\{y^{1}_{i},\ldots ,y^{n}_{i} \}$ and $\lambda^{j}_{i}\geq0, j=1,\ldots,n$, with $\sum_{j=1}^{n}\lambda^{j}_{i}=1$ such that

$$ y=\sum_{j=1}^{n} \lambda^{j}_{i} y^{j}_{i} \notin\bigcup_{j=1}^{n} Q_{i} \bigl(y^{j}_{i} \bigr). $$

Therefore, we have

$$\begin{aligned}& \rho_{i} N_{i} \bigl(z,\eta_{i} \bigl(f_{i} \bigl(y^{j}_{i} \bigr),f_{i}(y) \bigr) \bigr)+ \bigl\langle g_{i} \bigl(f_{i} \bigl(y_{i}^{j} \bigr) \bigr)-g_{i} \bigl(f_{i}(x_{i})\bigr) +\rho_{i} \bigl(M(u_{i},v_{i})+w_{i}\bigr),\eta_{i} \bigl(f_{i} \bigl(y^{j}_{i} \bigr),f_{i}(y) \bigr) \bigr\rangle \\& \quad{}+\rho _{i} \bigl\{ \phi_{i} \bigl(x_{i},y^{j}_{i} \bigr)- \phi_{i}(x_{i},y) \bigr\} < 0,\quad \forall i,j\text{ and } z\in A_{i}(y). \end{aligned}$$

Since $\eta_{i}$ and $f_{i}$ are affine, and $N_{i}$ and $\phi_{i}$ are convex in the second argument, we have

$$\begin{aligned} 0 =& \rho_{i} N_{i}\bigl(z,\eta_{i} \bigl(f_{i}(y),f_{i}(y)\bigr)\bigr)+ \bigl\langle g_{i} \bigl(f_{i} \bigl(y_{i}^{j} \bigr) \bigr)-g_{i}\bigl(f_{i}(x_{i})\bigr) + \rho_{i}\bigl(M(u_{i},v_{i})+w_{i} \bigr), \eta _{i}\bigl(f_{i}(y),f_{i}(y)\bigr) \bigr\rangle \\ &{}+\rho_{i}\bigl\{ \phi_{i}(x_{i},y)- \phi_{i}(x_{i},y)\bigr\} \\ =&\rho_{i} N_{i} \biggl(z,\eta_{i} \biggl(\sum _{j}\lambda^{j}_{i} f_{i} \bigl(y^{j}_{i} \bigr),f_{i}(y) \biggr) \biggr)+ \biggl\langle g_{i} \bigl(f_{i} \bigl(y_{i}^{j} \bigr) \bigr)-g_{i} \bigl(f_{i}(x_{i})\bigr) +\rho_{i} \bigl(M(u_{i},v_{i})+w_{i}\bigr), \\ &{}\eta_{i} \biggl(\sum_{j} \lambda^{j}_{i}f_{i} \bigl(y^{j}_{i} \bigr),f_{i}(y) \biggr) \biggr\rangle +\rho_{i} \biggl\{ \phi_{i} \biggl(x_{i},\sum_{j} \lambda^{j}_{i} y^{j}_{i} \biggr)- \phi_{i}(x_{i},y) \biggr\} \\ \leq&\rho_{i} N_{i} \biggl(z,\sum _{j}\lambda^{j}_{i}\eta_{i} \bigl(f_{i} \bigl(y^{j}_{i} \bigr),f_{i}(y) \bigr) \biggr)+ \biggl\langle g_{i} \bigl(f_{i} \bigl(y_{i}^{j} \bigr) \bigr)-g_{i} \bigl(f_{i}(x_{i})\bigr) +\rho_{i} \bigl(M(u_{i},v_{i})+w_{i}\bigr), \\ &{}\sum_{j}\lambda^{j}_{i} \eta_{i} \bigl(f_{i} \bigl(y^{j}_{i} \bigr),f_{i}(y_{i}) \bigr) \biggr\rangle +\rho_{i} \biggl\{ \sum_{j} \lambda^{j}_{i} \phi _{i} \bigl(x_{i},y^{j}_{i} \bigr)-\phi_{i}(x_{i},y) \biggr\} \\ \leq& \sum_{j}\lambda^{j}_{i} \bigl\{ \rho_{i} N_{i} \bigl(z,\eta_{i} \bigl(f_{i} \bigl(y^{j}_{i} \bigr),f_{i}(y) \bigr) \bigr) \bigr\} +\sum_{j}\lambda ^{j}_{i} \bigl\langle g_{i} \bigl(f_{i} \bigl(y_{i}^{j} \bigr) \bigr)-g_{i}\bigl(f_{i}(x_{i})\bigr)+ \rho_{i}\bigl(M(u_{i},v_{i}) \\ &{}+w_{i}\bigr),\eta_{i} \bigl(f_{i} \bigl(y^{j}_{i} \bigr),f_{i}(y) \bigr) \bigr\rangle +\rho_{i} \biggl\{ \sum_{j} \lambda^{j}_{i}\phi_{i} \bigl(x_{i}, y^{j}_{i} \bigr)-\sum_{j} \lambda^{j}_{i}\phi_{i}(x_{i},y) \biggr\} \\ =&\sum_{j}\lambda^{j}_{i} \bigl\{ \rho_{i} N_{i} \bigl(z,\eta_{i} \bigl(f_{i} \bigl(y^{j}_{i} \bigr),f_{i}(y) \bigr) \bigr)+ \bigl\langle g_{i} \bigl(f_{i} \bigl(y_{i}^{j} \bigr) \bigr)-g_{i} \bigl(f_{i}(x_{i})\bigr) +\rho_{i} \bigl(M(u_{i},v_{i})+w_{i}\bigr), \\ &{}\eta_{i} \bigl(f_{i} \bigl(y^{j}_{i} \bigr),f_{i}(y) \bigr) \bigr\rangle +\rho _{i} \bigl\{ \phi_{i} \bigl(x_{i},y^{j}_{i} \bigr)- \phi_{i}(x_{i},y) \bigr\} \bigr\} \\ < &0, \end{aligned}$$

which is a contradiction. Therefore, *y* being an arbitrary element of $\operatorname{Co} \{y^{1}_{i},\ldots,y^{n}_{i} \}$, we have $y \in \operatorname{Co} \{ y^{1}_{i},\ldots,y^{n}_{i} \}\subseteq\bigcup_{j=1}^{n} Q_{i} (y^{j}_{i} )$. Hence $Q_{i}$ is a KKM-mapping.

Now, we show that $\bigcap_{y_{i} \in K_{i}}Q_{i}(y_{i})=\bigcap_{y_{i} \in K_{i}}P_{i}(y_{i})$, for every $y_{i} \in K_{i}$. Let $t_{i} \in Q_{i}(y_{i})$, therefore by definition, we have

$$\begin{aligned}& \rho_{i} N_{i}\bigl(z_{i},\eta_{i} \bigl(f_{i}(y_{i}),f_{i}(t_{i})\bigr) \bigr)+\bigl\langle g_{i}\bigl(f_{i}(y_{i}) \bigr)-g_{i}\bigl(f_{i}(x_{i})\bigr) + \rho_{i}\bigl(M(u_{i},v_{i})+w_{i} \bigr), \eta _{i}\bigl(f_{i}(y_{i}),f_{i}(t_{i}) \bigr)\bigr\rangle \\& \quad{}+\rho_{i}\bigl\{ \phi_{i}(x_{i},y_{i})- \phi_{i}(x_{i},t_{i})\bigr\} \geq0, \end{aligned}$$

which implies that

7 $$\begin{aligned}& \bigl\langle g_{i}\bigl(f_{i}(y_{i}) \bigr),\eta_{i}\bigl(f_{i}(y_{i}),f_{i}(t_{i}) \bigr)\bigr\rangle +\rho_{i} N_{i}\bigl(z_{i}, \eta_{i}\bigl(f_{i}(t_{i}),f_{i}(y) \bigr)\bigr) \\& \quad\geq \bigl\langle g_{i}\bigl(f_{i}(x_{i}) \bigr) +\rho_{i}\bigl(M(u_{i},v_{i})+w_{i} \bigr), \eta _{i}\bigl(f_{i}(y_{i}),f_{i}(t_{i}) \bigr)\bigr\rangle +\rho_{i}\bigl\{ \phi_{i}(x_{i},y_{i})- \phi_{i}(x_{i},t_{i})\bigr\} . \end{aligned}$$

Since $g_{i}$ is $\varepsilon_{i}$-$\eta_{i}$-relaxed strongly monotone with respect to $f_{i}$ with the condition $\varepsilon_{i}=\rho_{i} \alpha_{i}$, inequality (7) becomes

$$\begin{aligned}& \rho_{i} N_{i}\bigl(z_{i},\eta_{i} \bigl(f_{i}(y_{i}),f_{i}(t_{i})\bigr) \bigr)+\bigl\langle g_{i}\bigl(f_{i}(t_{i}) \bigr)-g_{i}\bigl(f_{i}(x_{i})\bigr)+ \rho_{i} \bigl(M(u_{i},v_{i})+w_{i} \bigr),\eta_{i}\bigl(f_{i}(y_{i}),f_{i}(t_{i}) \bigr)\bigr\rangle \\& \quad{}+\rho_{i} \bigl\{ \phi_{i}(x_{i},y_{i})- \phi _{i}(x_{i},t_{i})+\alpha_{i} \bigl\| f_{i}(y_{i})-f_{i}(t_{i}) \bigr\| ^{2} \bigr\} \geq0, \end{aligned}$$

and hence we have $t_{i} \in P_{i}(y_{i})$. It follows that $\bigcap_{y_{i} \in K_{i}}Q_{i}(y_{i})\subseteq\bigcap_{y_{i} \in K_{i}}P_{i}(y_{i})$.

*Conversely*, suppose that $t_{i} \in\bigcap_{y_{i} \in K_{i}}P_{i}(y_{i})$, then we have

8 $$\begin{aligned}& \rho_{i} N_{i}\bigl(z_{i}, \eta_{i}\bigl(f_{i}(y_{i}),f_{i}(t_{i}) \bigr)\bigr) + \bigl\langle g_{i}\bigl(f_{i}(t_{i}) \bigr)-g_{i}\bigl(f_{i}(x_{i})\bigr)+ \rho_{i} \bigl\{ M(u_{i},v_{i})+w_{i} \bigr\} ,\eta_{i}\bigl(f_{i}(y_{i}),f_{i}(t_{i}) \bigr)\bigr\rangle \\& \quad{}+\rho_{i} \bigl\{ \phi_{i}(x_{i},y_{i})- \phi _{i}(x_{i},t_{i})+\alpha_{i} \bigl\| f_{i}(y_{i})-f_{i}(t_{i}) \bigr\| ^{2} \bigr\} \geq0. \end{aligned}$$

Let $y_{i}^{\lambda}=\lambda_{i} t_{i}+(1-\lambda_{i})y_{i}$, $\lambda_{i}\in [0,1]$. Since $K_{i}$ is convex, we have $y_{i}^{\lambda} \in K_{i}$. It follows from (8) that

9 $$\begin{aligned}& \rho_{i} N_{i} \bigl(z_{i}, \eta_{i} \bigl(f_{i} (y_{i} ),f_{i} \bigl(y_{i}^{\lambda} \bigr) \bigr) \bigr)+ \bigl\langle g_{i} \bigl(f_{i} \bigl(y_{i}^{\lambda} \bigr) \bigr)-g_{i}\bigl(f_{i}(x_{i})\bigr)+ \rho_{i} \bigl(M(u_{i},v_{i})+w_{i} \bigr),\eta_{i} \bigl(f_{i} (y_{i} ),f_{i} \bigl(y_{i}^{\lambda} \bigr) \bigr) \bigr\rangle \\& \quad{}+\rho_{i} \bigl\{ \phi_{i} (x_{i},y_{i} )-\phi_{i} \bigl(x_{i},y_{i}^{\lambda} \bigr)+\alpha_{i} \bigl\Vert f_{i} (y_{i} )-f_{i} \bigl(y_{i}^{\lambda} \bigr)\bigr\Vert ^{2} \bigr\} \geq0. \end{aligned}$$

Since $\eta_{i}$ is affine with the condition $\eta _{i}(f_{i}(y_{i}),f_{i}(y_{i}))=0$, $f_{i}$ is affine, and $N_{i}$ and $\phi_{i}$ are convex in the second argument, inequality (9) reduces to

$$\begin{aligned}& \lambda_{i} \rho_{i} N_{i} \bigl(z_{i},\eta_{i}\bigl(f_{i}(y_{i}),f_{i}(t_{i}) \bigr)\bigr)+\lambda_{i} \bigl\langle g_{i} \bigl(f_{i} \bigl(y_{i}^{\lambda} \bigr) \bigr)-g_{i}\bigl(f_{i}(x_{i})\bigr)+\rho _{i} \bigl(M(u_{i},v_{i})+w_{i} \bigr),\eta_{i}\bigl(f_{i}(y_{i}),f_{i}(t_{i}) \bigr) \bigr\rangle \\& \quad{}+\lambda_{i}\rho_{i} \bigl\{ \phi _{i}(x_{i},y_{i})-\phi_{i}(x_{i},t_{i})+ \alpha_{i}\lambda_{i}\bigl\| f_{i}(y_{i})-f_{i}(t_{i}) \bigr\| ^{2} \bigr\} \geq0, \end{aligned}$$

which implies that

10 $$\begin{aligned}& \lambda_{i} \rho_{i} N_{i} \bigl(z_{i},\eta_{i}\bigl(f_{i}(y_{i}),f_{i}(t_{i}) \bigr)\bigr)+\lambda_{i}\bigl\langle g_{i}\bigl( \lambda_{i} f_{i}(t_{i})+(1-\lambda_{i})f_{i}(y_{i}) \bigr)-g_{i}\bigl(f_{i}(x_{i})\bigr) \\ & \quad{}+\rho_{i} \bigl(M(u_{i},v_{i})+w_{i} \bigr),\eta_{i}\bigl(f_{i}(y_{i}),f_{i}(t_{i}) \bigr)\bigr\rangle +\lambda _{i}\rho_{i} \bigl\{ \phi_{i}(x_{i},y_{i})-\phi_{i}(x_{i},t_{i}) \\ & \quad{}+\alpha_{i} \lambda_{i} \bigl\| f_{i}(y_{i})-f_{i}(t_{i}) \bigr\| ^{2} \bigr\} \geq0. \end{aligned}$$

Dividing (10) by $\lambda_{i}$, we get

$$\begin{aligned}& \rho_{i} N_{i}\bigl(z_{i}, \eta_{i}\bigl(f_{i}(y_{i}),f_{i}(t_{i}) \bigr)\bigr)+\bigl\langle g_{i}\bigl(\lambda_{i} f_{i}(t_{i})+(1-\lambda_{i})f_{i}(y_{i}) \bigr)-g_{i}\bigl(f_{i}(x_{i})\bigr)+ \rho_{i} \bigl(M(u_{i},v_{i}) \\ & \quad{}+w_{i}\bigr),\eta_{i}\bigl(f_{i}(y_{i}),f_{i}(t_{i}) \bigr)\bigr\rangle +\rho_{i} \bigl\{ \phi _{i}(x_{i},y_{i})- \phi_{i}(x_{i},t_{i})+\alpha_{i} \lambda_{i} \bigl\| f_{i}(y_{i})-f_{i}(t_{i}) \bigr\| ^{2} \bigr\} \geq0. \end{aligned}$$

Since $g_{i}$ is hemicontinuous with respect to $f_{i}$ and taking $\lambda _{i} \rightarrow0$, it implies that

$$\begin{aligned}& \rho_{i} N_{i}\bigl(z_{i},\eta_{i} \bigl(f_{i}(y_{i}),f_{i}(t_{i})\bigr) \bigr)+\bigl\langle g_{i}\bigl(f_{i}(y_{i}) \bigr)-g_{i}\bigl(f_{i}(x_{i})\bigr)+ \rho_{i} \bigl(M(u_{i},v_{i})+w_{i} \bigr),\eta_{i}\bigl(f_{i}(y_{i}),f_{i}(t_{i}) \bigr)\bigr\rangle \\ & \quad{}+\rho_{i} \bigl\{ \phi_{i}(x_{i},y_{i})- \phi _{i}(x_{i},t_{i})\bigr\} \geq0. \end{aligned}$$

Therefore, we have $t_{i} \in Q_{i}(y_{i})$, and we conclude that $\bigcap_{y_{i} \in K_{i}}Q_{i}(y_{i})=\bigcap_{y_{i} \in K_{i}}P_{i}(y_{i})$ and $P_{i}$ is also a KKM-mapping, for each $y_{i} \in K_{i}$.

Since $\eta_{i}$ is continuous in the second argument, $f_{i}$ and $\phi_{i}$ are continuous and $N_{i}$ is upper semicontinuous, it follows that $P_{i}(y_{i})$ is closed for each $y_{i} \in K_{i}$.

Finally, we show that, for $t_{i}^{0} \in D_{i}\cap K_{i}$, $P_{i} (t_{i}^{0} )$ is compact. For this purpose, suppose that there exists $\tilde{t_{i}} \in P_{i} (t_{i}^{0} )$ such that $\tilde{t_{i}} \notin D$. Therefore, for $\tilde{z_{i}}\in A_{i} (\tilde{t}_{i} )$, we have

11 $$\begin{aligned}& \rho_{i} N_{i} \bigl(\tilde{z_{i}}, \eta_{i} \bigl(f_{i} \bigl(t_{i}^{0} \bigr),f_{i} (\tilde{t_{i}} ) \bigr) \bigr)+ \bigl\langle g_{i} \bigl(f_{i} \bigl(t_{i}^{0} \bigr) \bigr)-g_{i}\bigl(f_{i}(x_{i})\bigr)+ \rho_{i} \bigl(M_{i}(u_{i},v_{i})+w_{i} \bigr),\eta_{i} \bigl(f_{i} \bigl(t_{i}^{0} \bigr),f_{i} (\tilde{t_{i}} ) \bigr) \bigr\rangle \\ & \quad{}+ \rho_{i} \bigl\{ \phi_{i} \bigl(x_{i},t_{i}^{0} \bigr)-\phi_{i} (x_{i},\tilde{t_{i}} )+ \alpha_{i}\bigl\Vert f_{i} \bigl(t_{i}^{0} \bigr)-f_{i} (\tilde{t_{i}} )\bigr\Vert ^{2} \bigr\} \geq0. \end{aligned}$$

But by Assumption (viii), for $\tilde{t_{i}} \notin D$, we have

$$\begin{aligned}& \rho_{i} N_{i}\bigl(\tilde{z_{i}}, \eta_{i}\bigl(f_{i}\bigl(t_{i}^{0} \bigr),f_{i}(\tilde{t_{i}})\bigr)\bigr)+\bigl\langle g_{i}\bigl(f_{i}\bigl(t_{i}^{0}\bigr) \bigr)-g_{i}\bigl(f_{i}(x_{i})\bigr)+ \rho_{i} \bigl(M_{i}(u_{i},v_{i})+w_{i} \bigr),\eta_{i}\bigl(f_{i}\bigl(t_{i}^{0} \bigr),f_{i}(\tilde{t_{i}})\bigr)\bigr\rangle \\ & \quad {}+ \rho_{i}\bigl\{ \phi _{i}\bigl(x_{i},t_{i}^{0} \bigr)-\phi_{i}(x_{i},\tilde{t_{i}})\bigr\} + \rho_{i} \alpha_{i} \bigl\| f_{i}\bigl(t_{i}^{0} \bigr)-f_{i}(\tilde{t_{i}})\bigr\| ^{2} < 0, \end{aligned}$$

which is a contradiction to (11). Therefore $Q_{i}(t_{i})\subset D$. Due to compactness of *D*, and closedness of $P_{i} (t_{i}^{0} )$, we conclude that $P_{i} (t_{i}^{0} )$ is compact.

Thus, all the conditions of the Fan-KKM Theorem 2.1 are fulfilled by the mapping $P_{i}$. Therefore

$$ \bigcap_{y_{i} \in K_{i}} P_{i}(y_{i}) \neq\phi. $$

Hence, $(t_{1},t_{2}) \in K_{1} \times K_{2}$ is a solution of the perturbed system of auxiliary generalized multi-valued mixed quasi-equilibrium-like problems (6).

Now, let $(t_{1},t_{2}),(\tilde{t_{1}},\tilde{t_{2}})\in K_{1} \times K_{2}$ be any two solutions of the perturbed system of auxiliary generalized multi-valued mixed quasi-equilibrium-like problems (6). Then, for each $i \in I$, we have

12 $$\begin{aligned}& \rho_{i} N_{i} \bigl(\tilde{z_{i}}, \eta_{i} \bigl(f_{i}(y_{i}),f_{i} ( \tilde {t_{i}} ) \bigr) \bigr)+ \bigl\langle g_{i} \bigl(f_{i} (\tilde {t_{i}} ) \bigr)-g_{i} \bigl(f_{i}(x_{i})\bigr)+\rho_{i} \bigl(M_{i}(u_{i},v_{i})+w_{i}\bigr),\eta_{i} \bigl(f_{i}(y_{i}),f_{i} (\tilde{t_{i}} ) \bigr) \bigr\rangle \\ & \quad {}+\rho_{i} \bigl\{ \phi_{i}(x_{i},y_{i})-\phi_{i} (x_{i},\tilde{t_{i}} )+\alpha _{i} \bigl\Vert f_{i}(y_{i})-f_{i} (\tilde{t_{i}} ) \bigr\Vert ^{2} \bigr\} \geq 0 \end{aligned}$$

and

13 $$\begin{aligned}& \rho_{i} N_{i} \bigl(z_{i}, \eta_{i} \bigl(f_{i}(y_{i}),f_{i} (t_{i} ) \bigr) \bigr)+ \bigl\langle g_{i} \bigl(f_{i} (t_{i} ) \bigr)-g_{i} \bigl(f_{i}(x_{i})\bigr)+\rho_{i} \bigl(M_{i}(u_{i},v_{i})+w_{i}\bigr),\eta_{i} \bigl(f_{i}(y_{i}),f_{i} (t_{i} ) \bigr) \bigr\rangle \\ & \quad{}+\rho _{i} \bigl\{ \phi_{i}(x_{i},y_{i})-\phi_{i} (x_{i},t_{i} )+\alpha_{i} \bigl\Vert f_{i}(y_{i})-f_{i} (t_{i} )\bigr\Vert ^{2} \bigr\} \geq0. \end{aligned}$$

Putting $y_{i} =t_{i}$ in (12) and $y_{i}=\tilde{t_{i}}$ in (13), summing up the resulting inequalities and using the condition $\eta_{i}(f_{i}(x_{i}),f_{i}(y_{i}))+ \eta_{i} (f_{i}(y_{i}),f_{i}(x_{i}))=0$, we have

14 $$\begin{aligned}& \rho_{i} \bigl\{ N_{i} \bigl( \tilde{z_{i}},\eta_{i} \bigl(f_{i}(t_{i}),f_{i} (\tilde{t_{i}} ) \bigr) \bigr)+ N_{i} \bigl(z_{i}, \eta_{i} \bigl(f_{i} (\tilde{t_{i}} ),f_{i}(t_{i}) \bigr) \bigr) \bigr\} + \bigl\langle g_{i} \bigl(f_{i} (\tilde{t_{i}} ) \bigr)-g_{i}\bigl(f_{i}(t_{i})\bigr),\eta_{i} \bigl(f_{i}(t_{i}),f_{i} (\tilde{t_{i}} ) \bigr) \bigr\rangle \\ & \quad{}+2\rho_{i} \alpha_{i}\bigl\Vert f_{i}(t_{i})-f_{i} (\tilde{t_{i}} )\bigr\Vert ^{2}\geq0. \end{aligned}$$

Since $N_{i}$ is strongly $\varrho_{i}$-$\eta_{i}$-$f_{i}$-strongly monotone with respect to $A_{i}$, $g_{i}$ is $\varepsilon_{i}$-$\eta_{i}$-relaxed strongly monotone with respect to $f_{i}$ with the condition $\varepsilon _{i}=\alpha_{i} \rho_{i}$, we have from (14)

$$\begin{aligned}& -\rho_{i}\varrho_{i}\bigl\Vert f_{i}(t_{i})-f_{i} (\tilde{t_{i}} )\bigr\Vert ^{2}+2\rho_{i} \alpha_{i}\bigl\Vert f_{i}(t_{i})-f_{i} (\tilde{t_{i}} )\bigr\Vert ^{2} \\ & \quad\geq \rho_{i} \bigl\{ N_{i} \bigl(\tilde{z_{i}}, \eta_{i} \bigl(f_{i}(t_{i}),f_{i} ( \tilde{t_{i}} ) \bigr) \bigr)+ N_{i} \bigl(z_{i}, \eta_{i} \bigl(f_{i} (\tilde{t_{i}} ),f_{i}(t_{i}) \bigr) \bigr) \bigr\} +2\rho_{i} \alpha_{i}\bigl\Vert f_{i}(t_{i})-f_{i} (\tilde{t_{i}} )\bigr\Vert ^{2} \\ & \quad\geq \bigl\langle g_{i} \bigl(f_{i} ( \tilde{t_{i}} ) \bigr)-g_{i}\bigl(f_{i}(t_{i}) \bigr),\eta_{i} \bigl(f_{i}(t_{i}),f_{i} (\tilde{t_{i}} ) \bigr) \bigr\rangle \\ & \quad\geq -\varepsilon_{i}\bigl\Vert f_{i}(t_{i})-f_{i} (\tilde{t_{i}} )\bigr\Vert ^{2}, \end{aligned}$$

which implies that

$$ (-\rho_{i}\varrho_{i}+3\varepsilon_{i})\bigl\Vert f_{i}(t_{i})-f_{i} (\tilde {t_{i}} )\bigr\Vert ^{2}\geq0. $$

Since $f_{i}$ is $\beta_{i}$-expansive and $3\varepsilon_{i}<\rho_{i}\varrho _{i}$, we obtain

$$ 0\leq(-\rho_{i}\varrho_{i}+3\varepsilon_{i}) \bigl\Vert f_{i}(t_{i})-f_{i} (\tilde {t_{i}} )\bigr\Vert ^{2}\leq(-\rho_{i} \varrho_{i}+3\varepsilon_{i})\beta _{i}^{2} \Vert t_{i}-\tilde{t_{i}}\Vert ^{2}< 0, $$

which shows that $\tilde{t_{i}}=t_{i}$. This completes the proof. □

## Iterative algorithm and convergence analysis

By using Theorem 3.1 and Lemma 2.1, we construct the following iterative algorithm for computing approximate solutions of the perturbed system of generalized multi-valued mixed quasi-equilibrium-like problems (1).

### Iterative Algorithm 4.1

For any given $(x_{1}^{0}, x_{2}^{0} )\in K_{1}\times K_{2}$, $u_{1}^{0} \in T_{1} (x_{1}^{0} )$, $u_{2}^{0} \in T_{2} (x_{1}^{0} )$, $v_{1}^{0} \in S_{1} (x_{2}^{0} )$, $v_{2}^{0} \in S_{1} (x_{2}^{0} )$, $w_{1}^{0} \in B_{1} (x_{1}^{0},x_{2}^{0} )$, $w_{2}^{0} \in B_{2} (x_{1}^{0},x_{2}^{0} )$ and $z_{1}^{0} \in A_{1} (x_{1}^{0} )$, $z_{2}^{0} \in A_{2} (x_{2}^{0} )$, compute the iterative sequences $\{ (x_{1}^{n},x_{2}^{n} ) \} \subseteq K_{1} \times K_{2}$, $\{u_{i}^{n} \}$, $\{ v_{i}^{n} \}$, $\{w_{i}^{n} \}$ and $\{z_{i}^{n} \}$ by the following iterative schemes:

15 $$\begin{aligned}& \rho_{1} N_{1} \bigl(z_{1}^{n+1}, \eta_{1} \bigl(f_{1}(y_{1}),f_{1} \bigl(x_{1}^{n+1} \bigr) \bigr) \bigr)+ \bigl\langle g_{1} \bigl(f_{1} \bigl(x_{1}^{n+1} \bigr) \bigr)-g_{1} \bigl(f_{1} \bigl(x_{1}^{n} \bigr) \bigr) + \rho _{1} \bigl\{ M_{1} \bigl(u_{1}^{n},v_{1}^{n} \bigr) \\ & \quad{}+w_{1}^{n} \bigr\} ,\eta_{1} \bigl(f_{1}(y_{1}),f_{1} \bigl(x_{1}^{n+1} \bigr) \bigr) \bigr\rangle + \rho_{1} \bigl\{ \phi_{1} \bigl(x_{1}^{n},y_{1} \bigr)-\phi_{1} \bigl(x_{1}^{n},x_{1}^{n+1} \bigr) \\& \quad{}+\alpha_{1} \bigl\Vert f_{1}(y_{1})-f_{1} \bigl(x_{1}^{n+1} \bigr)\bigr\Vert ^{2} \bigr\} \geq0,\quad\forall y_{1} \in K_{1}; \end{aligned}$$

16 $$\begin{aligned}& \rho_{2} N_{2} \bigl(z_{2}^{n+1}, \eta_{2} \bigl(f_{2}(y_{2}),f_{2} \bigl(x_{2}^{n+1} \bigr) \bigr) \bigr)+ \bigl\langle g_{2} \bigl(f_{2} \bigl(x_{2}^{n+1} \bigr) \bigr)-g_{2} \bigl(f_{2} \bigl(x_{2}^{n} \bigr) \bigr) + \rho _{2} \bigl\{ M_{2} \bigl(u_{2}^{n},v_{2}^{n} \bigr) \\ & \quad{}+w_{2}^{n} \bigr\} ,\eta_{2} \bigl(f_{2}(y_{2}),f_{2} \bigl(x_{2}^{n+1} \bigr) \bigr) \bigr\rangle + \rho_{2} \bigl\{ \phi_{2} \bigl(x_{2}^{n},y_{2} \bigr)-\phi_{2} \bigl(x_{2}^{n},x_{2}^{n+1} \bigr) \\ & \quad{}+\alpha_{2} \bigl\Vert f_{2}(y_{2})-f_{2} \bigl(x_{2}^{n+1} \bigr)\bigr\Vert ^{2} \bigr\} \geq0,\quad\forall y_{2} \in K_{2}; \end{aligned}$$

17 $$\begin{aligned}& \textstyle\begin{cases} u_{i}^{n} \in T_{i} (x_{1}^{n} ); & \Vert u_{i}^{n+1}-u_{i}^{n}\Vert \leq (1+\frac{1}{n+1} )\mathcal{D} (T_{i} (x_{1}^{n+1} ),T_{i} (x_{1}^{n} ) );\\ v_{i}^{n} \in S_{i} (x_{2}^{n} ); & \Vert v_{i}^{n+1}-v_{i}^{n}\Vert \leq (1+\frac{1}{n+1} )\mathcal{D} (S_{i} (x_{2}^{n+1} ),S_{i} (x_{2}^{n} ) );\\ w_{i}^{n} \in B_{i} (x_{1}^{n}, x_{2}^{n} ); & \Vert w_{i}^{n+1}-w_{i}^{n}\Vert \leq (1+\frac{1}{n+1} )\mathcal {D} (B_{i} (x_{1}^{n+1},x_{2}^{n+1} ),B_{i} (x_{1}^{n},x_{2}^{n} ) );\\ z_{i}^{n} \in A_{i} (x_{1}^{n} ); & \Vert z_{i}^{n+1}-z_{i}^{n}\Vert \leq (1+\frac{1}{n+1} )\mathcal{D} (A_{i} (x_{i}^{n+1} ),A_{i} (x_{i}^{n} ) ), \end{cases}\displaystyle \end{aligned}$$

where $n=0,1,2,\ldots$ , $i=1,2$, and $\rho_{1}, \rho_{2}, \alpha_{1}, \alpha _{2}>0$ are constants.

Now, we establish the following strong convergence result to obtain the solution of perturbed system of generalized multi-valued mixed quasi-equilibrium-like problems (1).

### Theorem 4.1

*For each* $i\in I$, *the mappings* $N_{i}$, $M_{i}$, $A_{i}$, $T_{i}$, $S_{i}$, $B_{i}$, $\eta_{i}$, $\phi_{i}$, *and* $f_{i}$ *satisfy the hypotheses of Theorem* 3.1. *Further assume that*:

(i) $M_{i}$ *is* $(\mu_{i},\xi_{i} )$-*mixed Lipschitz continuous*;
(ii) $g_{i}$ *is* $\sigma_{i}$-*Lipschitz continuous with respect to* $f_{i}$ *and* $\eta_{i}$ *is* $\kappa_{i}$-*Lipschitz continuous with respect to* $f_{i}$;
(iii) $T_{i}$ *is* $\delta_{i}$-$\mathcal{D}$-*Lipschitz continuous and* $S_{i}$ *is* $\tau_{i}$-$\mathcal{D}$-*Lipschitz continuous*;
(iv) $B_{i}$ *is* $(\zeta_{i},\nu_{i} )$-$\mathcal {D}$-*Lipschitz continuous and* $A_{i}$ *is* $\varsigma_{i}$-$\mathcal {D}$-*Lipschitz continuous*.

*For* $\rho_{1}, \rho_{2}>0$, *if the following conditions are satisfied*:

18 $$\begin{aligned} \textstyle\begin{cases} \frac{1}{(\rho_{1} \varrho_{1}-3 \varepsilon_{1})\beta_{1}^{2}} \{\kappa _{1}\sigma_{1}+\rho_{1}\kappa_{1} (\mu_{1}\delta_{1}+\zeta_{1} )+\rho_{1} \gamma_{1} \}+\frac{1}{(\rho_{2} \varrho_{2}-3 \varepsilon_{2})\beta _{2}^{2}} \{\rho_{2}\kappa_{2} (\mu_{2}\delta_{2}+\zeta_{2} ) \} < 1,\\ \frac{1}{(\rho_{2}\varrho_{2}-3 \varepsilon_{2})\beta_{2}^{2}} \{\kappa _{2}\sigma_{2}+\rho_{2}\kappa_{2} (\xi_{2}\tau_{2}+\nu_{2} )+\rho_{2}\gamma _{2} \}+\frac{1}{(\rho_{1} \varrho_{1}-3 \varepsilon_{1})\beta_{1}^{2}} \{ \rho_{1}\kappa_{1} (\xi_{1}\tau_{1}+\nu_{1} ) \}< 1, \end{cases}\displaystyle \end{aligned}$$

*then there exist* $(x_{1},x_{2})\in K_{1}\times K_{2}$, $u_{i}\in T_{i}(x_{1})$, $v_{i}\in S_{i}(x_{2})$, $w_{i}\in B_{i}(x_{1},x_{2})$, *and* $z_{i}\in A_{i}(x_{i})$ *such that* $(x_{1},x_{2},u_{1},u_{2},v_{1},v_{2},w_{1},w_{2},z_{1},z_{2} )$ *is the solution of the perturbed system of generalized multi*-*valued mixed quasi*-*equilibrium*-*like problems* (1) *and the sequences* $\{ x_{1}^{n} \}$, $\{x_{2}^{n} \}$, $\{u_{i}^{n} \}$, $\{v_{i}^{n} \}$, $\{w_{i}^{n} \}$, *and* $\{ z_{i}^{n} \}$ *generated by Algorithm* 4.1 *converge strongly to* $x_{1}$, $x_{2}$, $u_{i}$, $v_{i}$, $w_{i}$, *and* $z_{i}$, *respectively*.

### Proof

Firstly, from (15) of Algorithm 4.1, we have, for all $y_{1} \in K_{1}$,

19 $$\begin{aligned}& \rho_{1} N_{1} \bigl(z_{1}^{n}, \eta_{1} \bigl(f_{1}(y_{1}),f_{1} \bigl(x_{1}^{n} \bigr) \bigr) \bigr)+ \bigl\langle g_{1} \bigl(f_{1} \bigl(x_{1}^{n} \bigr) \bigr)-g_{1} \bigl(f_{1} \bigl(x_{1}^{n-1} \bigr) \bigr) + \rho _{1} \bigl\{ M_{1} \bigl(u_{1}^{n-1},v_{1}^{n-1} \bigr) \\ & \quad{}+w_{1}^{n-1} \bigr\} ,\eta_{1} \bigl(f_{1}(y_{1}),f_{1} \bigl(x_{1}^{n} \bigr) \bigr) \bigr\rangle +\rho_{1} \bigl\{ \phi_{1} \bigl(x_{1}^{n-1},y_{1} \bigr)-\phi_{1} \bigl(x_{1}^{n-1},x_{1}^{n} \bigr) \\ & \quad{}+\alpha_{1} \bigl\Vert f_{1}(y_{1})-f_{1} \bigl(x_{1}^{n} \bigr)\bigr\Vert ^{2} \bigr\} \geq0 \end{aligned}$$

and

20 $$\begin{aligned}& \rho_{1} N_{1} \bigl(z_{1}^{n+1}, \eta_{1} \bigl(f_{1}(y_{1}),f_{1} \bigl(x_{1}^{n+1} \bigr) \bigr) \bigr)+ \bigl\langle g_{1} \bigl(f_{1} \bigl(x_{1}^{n+1} \bigr) \bigr)-g_{1} \bigl(f_{1} \bigl(x_{1}^{n} \bigr) \bigr) + \rho _{1} \bigl\{ M_{1} \bigl(u_{1}^{n},v_{1}^{n} \bigr) \\ & \quad{}+w_{1}^{n} \bigr\} ,\eta_{1} \bigl(f_{1}(y_{1}),f_{1} \bigl(x_{1}^{n+1} \bigr) \bigr) \bigr\rangle +\rho_{1} \bigl\{ \phi_{1} \bigl(x_{1}^{n},y_{1} \bigr)-\phi_{1} \bigl(x_{1}^{n},x_{1}^{n+1} \bigr) \\ & \quad{}+\alpha_{1} \bigl\Vert f_{1}(y_{1})-f_{1} \bigl(x_{1}^{n+1} \bigr)\bigr\Vert ^{2} \bigr\} \geq0. \end{aligned}$$

Putting $y_{1}=x_{1}^{n+1}$ in (19) and $y_{1}=x_{1}^{n}$ in (20), and summing up the resulting inequalities, we obtain

$$\begin{aligned}& \rho_{1} \bigl\{ N_{1} \bigl(z_{1}^{n}, \eta_{1} \bigl(f_{1} \bigl(x_{1}^{n+1} \bigr),f_{1} \bigl(x_{1}^{n} \bigr) \bigr) \bigr)+N_{1} \bigl(z_{1}^{n+1}, \eta_{1} \bigl(f_{1} \bigl(x_{1}^{n} \bigr),f_{1} \bigl(x_{1}^{n+1} \bigr) \bigr) \bigr) \bigr\} \\& \quad{}+\bigl\langle g_{1} \bigl(f_{1} \bigl(x_{1}^{n} \bigr) \bigr)-g_{1} \bigl(f_{1} \bigl(x_{1}^{n-1} \bigr) \bigr)+\rho_{1} \bigl\{ M_{1} \bigl(u_{1}^{n-1},v_{1}^{n-1} \bigr)+w_{1}^{n-1} \bigr\} ,\eta_{1} \bigl(f_{1} \bigl(x_{1}^{n+1} \bigr),f_{1} \bigl(x_{1}^{n} \bigr) \bigr) \bigr\rangle \\& \quad{}+ \bigl\langle g_{1} \bigl(f_{1} \bigl(x_{1}^{n+1} \bigr) \bigr)-g_{1} \bigl(f_{1} \bigl(x_{1}^{n} \bigr) \bigr)+\rho_{1} \bigl\{ M_{1} \bigl(u_{1}^{n},v_{1}^{n} \bigr)+w_{1}^{n} \bigr\} ,\eta_{1} \bigl(f_{1} \bigl(x_{1}^{n} \bigr),f_{1} \bigl(x_{1}^{n+1} \bigr) \bigr) \bigr\rangle \\& \quad{}+\rho_{1} \bigl\{ \phi_{1} \bigl(x_{1}^{n-1},x_{1}^{n+1} \bigr)-\phi_{1} \bigl(x_{1}^{n-1},x_{1}^{n} \bigr)+\phi_{1} \bigl(x_{1}^{n},x_{1}^{n} \bigr)-\phi_{1} \bigl(x_{1}^{n},x_{1}^{n+1} \bigr) \\& \quad{}+\alpha_{1}\bigl\Vert f_{1} \bigl(x_{1}^{n+1} \bigr)-f_{1} \bigl(x_{1}^{n} \bigr)\bigr\Vert ^{2}+\alpha_{1}\bigl\Vert f_{1} \bigl(x_{1}^{n} \bigr)-f_{1} \bigl(x_{1}^{n+1} \bigr)\bigr\Vert ^{2} \bigr\} \geq0, \end{aligned}$$

which implies that

$$\begin{aligned}& \bigl\langle g_{1} \bigl(f_{1} \bigl(x_{1}^{n-1} \bigr) \bigr)-g_{1} \bigl(f_{1} \bigl(x_{1}^{n} \bigr) \bigr),\eta_{1} \bigl(f_{1} \bigl(x_{1}^{n} \bigr),f_{1} \bigl(x_{1}^{n+1} \bigr) \bigr) \bigr\rangle \\ & \qquad{}+\rho_{1} \bigl\langle M_{1} \bigl(u_{1}^{n},v_{1}^{n} \bigr),\eta_{1} \bigl(f_{1} \bigl(x_{1}^{n} \bigr),f_{1} \bigl(x_{1}^{n+1} \bigr) \bigr) \bigr\rangle +2\alpha_{1}\rho_{1}\bigl\Vert f_{1} \bigl(x_{1}^{n+1} \bigr)-f_{1} \bigl(x_{1}^{n} \bigr)\bigr\Vert ^{2} \\ & \qquad{}+\rho_{1} \bigl\langle w_{1}^{n}-w_{1}^{n-1}, \eta_{1} \bigl(f_{1} \bigl(x_{1}^{n} \bigr),f_{1} \bigl(x_{1}^{n+1} \bigr) \bigr) \bigr\rangle +\rho_{1}\phi_{1} \bigl(x_{1}^{n}-x_{1}^{n-1},x_{1}^{n}-x_{1}^{n+1} \bigr) \\ & \quad\geq \rho_{1} \bigl\{ N_{1} \bigl(z_{1}^{n}, \eta_{1} \bigl(f_{1} \bigl(x_{1}^{n+1} \bigr),f_{1} \bigl(x_{1}^{n} \bigr) \bigr) \bigr)+N_{1} \bigl(z_{1}^{n+1}, \eta_{1} \bigl(f_{1} \bigl(x_{1}^{n} \bigr),f_{1} \bigl(x_{1}^{n+1} \bigr) \bigr) \bigr) \bigr\} \\ & \qquad{}+ \bigl\langle g_{1} \bigl(f_{1} \bigl(x_{1}^{n} \bigr) \bigr)-g_{1} \bigl(f_{1} \bigl(x_{1}^{n+1} \bigr) \bigr),\eta_{1} \bigl(f_{1} \bigl(x_{1}^{n} \bigr),f_{1} \bigl(x_{1}^{n+1} \bigr) \bigr) \bigr\rangle . \end{aligned}$$

Since $N_{1}$ is $\varrho_{1}$-$\eta_{1}$-$f_{1}$-strongly monotone with respect to $A_{1}$, $g_{1}$ is $\varepsilon_{1}$-$\eta_{1}$-relaxed strongly monotone with respect to $f_{1}$, $\phi_{1}$ is bounded by assumption and using the Cauchy-Schwartz inequality, we have

21 $$\begin{aligned}& \rho_{1} \varrho_{1} \bigl\Vert f_{1} \bigl(x_{1}^{n+1} \bigr)-f_{1} \bigl(x_{1}^{n} \bigr)\bigr\Vert ^{2}- \varepsilon_{1} \bigl\Vert f_{1} \bigl(x_{1}^{n+1} \bigr)-f_{1} \bigl(x_{1}^{n} \bigr)\bigr\Vert ^{2} \\ & \quad\leq \rho_{1} \bigl\{ N_{1} \bigl(z_{1}^{n}, \eta_{1} \bigl(f_{1} \bigl(x_{1}^{n+1} \bigr),f_{1} \bigl(x_{1}^{n} \bigr) \bigr) \bigr)+N_{1} \bigl(z_{1}^{n+1}, \eta_{1} \bigl(f_{1} \bigl(x_{1}^{n} \bigr),f_{1} \bigl(x_{1}^{n+1} \bigr) \bigr) \bigr) \bigr\} \\ & \qquad {}+\bigl\langle g_{1} \bigl(f_{1} \bigl(x_{1}^{n} \bigr) \bigr)-g_{1} \bigl(f_{1} \bigl(x_{1}^{n+1} \bigr) \bigr),\eta_{1} \bigl(f_{1} \bigl(x_{1}^{n} \bigr),f_{1} \bigl(x_{1}^{n+1} \bigr) \bigr) \bigr\rangle \\ & \quad\leq\bigl\Vert g_{1} \bigl(f_{1} \bigl(x_{1}^{n-1} \bigr) \bigr)-g_{1} \bigl(f_{1} \bigl(x_{1}^{n} \bigr) \bigr)\bigr\Vert \bigl\Vert \eta_{1} \bigl(f_{1} \bigl(x_{1}^{n} \bigr),f_{1} \bigl(x_{1}^{n+1} \bigr) \bigr)\bigr\Vert \\ & \qquad{}+\rho_{1}\bigl\Vert M_{1} \bigl(u_{1}^{n},v_{1}^{n} \bigr)-M_{1} \bigl(u_{1}^{n-1},v_{1}^{n-1} \bigr)\bigr\Vert \bigl\Vert \eta_{1} \bigl(f_{1} \bigl(x_{1}^{n} \bigr),f_{1} \bigl(x_{1}^{n+1} \bigr) \bigr)\bigr\Vert \\ & \qquad {}+\rho_{1}\bigl\Vert w_{1}^{n}-w_{1}^{n-1} \bigr\Vert \bigl\Vert \eta_{1} \bigl(f_{1} \bigl(x_{1}^{n} \bigr),f_{1} \bigl(x_{1}^{n+1} \bigr) \bigr)\bigr\Vert + \rho_{1}\gamma _{1} \bigl\Vert x_{1}^{n}-x_{1}^{n-1}\bigr\Vert \bigl\Vert x_{1}^{n}-x_{1}^{n+1}\bigr\Vert \\ & \qquad{}+2\alpha_{1}\rho_{1}\bigl\Vert f_{1} \bigl(x_{1}^{n+1} \bigr)-f_{1} \bigl(x_{1}^{n} \bigr)\bigr\Vert ^{2}. \end{aligned}$$

By using $(\mu_{1},\xi_{1} )$-mixed Lipschitz continuity of $M_{1}$, $\delta_{i}$-$\mathcal{D}$-Lipschitz continuity of $T_{1}$ and $\tau _{i}$-$\mathcal{D}$-Lipschitz continuity of $S_{1}$, it follows by Algorithm 4.1 that

22 $$\begin{aligned}& \bigl\Vert M_{1} \bigl(u_{1}^{n},v_{1}^{n} \bigr)-M_{1} \bigl(u_{1}^{n-1},v_{1}^{n-1} \bigr)\bigr\Vert \\& \quad\leq\mu_{1}\bigl\Vert u_{1}^{n}-u_{1}^{n-1} \bigr\Vert +\xi_{1}\bigl\Vert v_{1}^{n}-v_{1}^{n-1} \bigr\Vert \\& \quad\leq\mu_{1} \biggl(1+\frac{1}{n} \biggr)\mathcal{D}\bigl\Vert T_{1} \bigl(x_{1}^{n} \bigr)-T_{1} \bigl(x_{1}^{n-1} \bigr)\bigr\Vert +\xi_{1} \biggl(1+\frac {1}{n} \biggr) \mathcal{D}\bigl\Vert S_{1} \bigl(x_{2}^{n} \bigr)-S_{1} \bigl(x_{2}^{n-1} \bigr)\bigr\Vert \\& \quad\leq\mu_{1}\delta_{1} \biggl(1+\frac{1}{n} \biggr) \bigl\Vert x_{1}^{n}-x_{1}^{n-1}\bigr\Vert +\xi_{1}\tau_{1} \biggl(1+\frac{1}{n} \biggr) \bigl\Vert x_{2}^{n}-x_{2}^{n-1}\bigr\Vert . \end{aligned}$$

Also by Algorithm 4.1 and $(\zeta_{1},\nu_{1} )$-$\mathcal{D}$-Lipschitz continuity of $B_{1}$, we have

23 $$\begin{aligned} \bigl\Vert w_{1}^{n}-w_{1}^{n-1} \bigr\Vert \leq& \biggl(1+\frac{1}{n} \biggr)\mathcal {D} \bigl(B_{1} \bigl(x_{1}^{n},x_{2}^{n} \bigr),B_{1} \bigl(x_{1}^{n-1},x_{2}^{n-1} \bigr) \bigr) \\ \leq& \biggl(1+\frac{1}{n} \biggr) \bigl(\zeta_{1}\bigl\Vert x_{1}^{n}-x_{1}^{n-1}\bigr\Vert + \nu_{1}\bigl\Vert x_{2}^{n}-x_{2}^{n-1} \bigr\Vert \bigr) \\ =&\zeta_{1} \biggl(1+\frac{1}{n} \biggr)\bigl\Vert x_{1}^{n}-x_{1}^{n-1}\bigr\Vert +\nu _{1} \biggl(1+\frac{1}{n} \biggr)\bigl\Vert x_{2}^{n}-x_{2}^{n-1}\bigr\Vert . \end{aligned}$$

Since $g_{1}$ is $\sigma_{1}$-Lipschitz continuous with respect to $f_{1}$, $\eta_{1}$ is $\kappa_{1}$-Lipschitz continuous with respect to $f_{1}$, $f_{1}$ is $\beta_{1}$-expansive with the condition $3\varepsilon_{1}<\rho _{1}\varrho_{1}$, it follows from (21), (22), and (23) that

$$\begin{aligned}& (\rho_{1} \varrho_{1}-3 \varepsilon_{1} ) \beta_{1}^{2} \bigl\Vert x_{1}^{n+1}-x_{1}^{n} \bigr\Vert ^{2} \\& \quad\leq (\rho_{1}\varrho_{1}-3\varepsilon_{1})\bigl\Vert f_{1} \bigl(x_{1}^{n+1} \bigr)-f_{1} \bigl(x_{1}^{n} \bigr)\bigr\Vert ^{2} \\& \quad\leq \kappa_{1} \sigma_{1} \bigl\Vert x_{1}^{n}-x_{1}^{n-1}\bigr\Vert \bigl\Vert x_{1}^{n+1}-x_{1}^{n}\bigr\Vert +\rho_{1}\kappa_{1} \biggl\{ \mu_{1} \delta_{1} \biggl(1+\frac{1}{n} \biggr) \bigl\Vert x_{1}^{n}-x_{1}^{n-1}\bigr\Vert \\& \qquad{}+ \xi_{1} \tau_{1}\biggl(1+\frac{1}{n} \biggr)\bigl\Vert x_{2}^{n}-x_{2}^{n-1} \bigr\Vert \biggr\} \bigl\Vert x_{1}^{n+1}-x_{1}^{n} \bigr\Vert +\kappa_{1}\rho_{1} \biggl\{ \zeta_{1} \biggl(1+\frac {1}{n} \biggr) \bigl\Vert x_{1}^{n}-x_{1}^{n-1} \bigr\Vert \\& \qquad{}+\nu_{1} \biggl(1+\frac{1}{n} \biggr) \bigl\Vert x_{2}^{n}-x_{2}^{n-1}\bigr\Vert \biggr\} \bigl\Vert x_{1}^{n+1}-x_{1}^{n}\bigr\Vert +\rho_{1} \gamma_{1} \bigl\Vert x_{1}^{n}-x_{1}^{n-1} \bigr\Vert \bigl\Vert x_{1}^{n+1}-x_{1}^{n} \bigr\Vert \\& \quad= \kappa_{1}\sigma_{1} \bigl\Vert x_{1}^{n}-x_{1}^{n-1} \bigr\Vert \bigl\Vert x_{1}^{n+1}-x_{1}^{n} \bigr\Vert +\rho_{1}\kappa_{1}\mu_{1} \delta_{1} \biggl(1+\frac {1}{n} \biggr) \bigl\Vert x_{1}^{n}-x_{1}^{n-1}\bigr\Vert \bigl\Vert x_{1}^{n+1}-x_{1}^{n}\bigr\Vert \\& \qquad{}+\rho_{1}\kappa_{1}\xi_{1} \tau_{1} \biggl(1+\frac{1}{n} \biggr)\bigl\Vert x_{2}^{n}-x_{2}^{n-1} \bigr\Vert \bigl\Vert x_{1}^{n+1}-x_{1}^{n} \bigr\Vert +\kappa_{1}\rho _{1}\zeta_{1} \biggl(1+ \frac{1}{n} \biggr) \bigl\Vert x_{1}^{n}-x_{1}^{n-1} \bigr\Vert \bigl\Vert x_{1}^{n+1} \\& \qquad{}-x_{1}^{n} \bigr\Vert +\kappa_{1}\rho_{1} \nu_{1} \biggl(1+ \frac{1}{n} \biggr)\bigl\Vert x_{2}^{n}-x_{2}^{n-1} \bigr\Vert \bigl\Vert x_{1}^{n+1}-x_{1}^{n} \bigr\Vert +\rho_{1} \gamma_{1} \bigl\Vert x_{1}^{n}-x_{1}^{n-1}\bigr\Vert \bigl\Vert x_{1}^{n+1}-x_{1}^{n}\bigr\Vert \\& \quad= \biggl\{ \kappa_{1}\sigma_{1}+\rho_{1} \kappa_{1}\mu_{1}\delta_{1} \biggl(1+ \frac {1}{n} \biggr)+\kappa_{1}\rho_{1} \zeta_{1} \biggl(1+\frac{1}{n} \biggr)+\rho_{1} \gamma_{1} \biggr\} \bigl\Vert x_{1}^{n}-x_{1}^{n-1} \bigr\Vert \bigl\Vert x_{1}^{n+1}-x_{1}^{n} \bigr\Vert \\& \qquad {}+\biggl\{ \rho_{1}\kappa_{1}\xi_{1} \tau_{1} \biggl(1+\frac{1}{n} \biggr)+\kappa _{1} \rho_{1} \nu_{1} \biggl(1+\frac{1}{n} \biggr) \biggr\} \bigl\Vert x_{2}^{n}-x_{2}^{n-1}\bigr\Vert \bigl\Vert x_{1}^{n+1}-x_{1}^{n} \bigr\Vert , \end{aligned}$$

which implies that

$$\begin{aligned}& \bigl\Vert x_{1}^{n+1}-x_{1}^{n}\bigr\Vert \\& \quad\leq \frac{1}{ (\rho_{1}\varrho_{1}-3 \varepsilon_{1} )\beta _{1}^{2}} \biggl[ \biggl\{ \kappa_{1} \sigma_{1}+ \biggl(\rho_{1}\kappa_{1} \biggl(1+ \frac {1}{n} \biggr) \biggr) (\mu_{1}\delta_{1}+ \zeta_{1} )+\rho_{1} \gamma _{1} \biggr\} \bigl\Vert x_{1}^{n}-x_{1}^{n-1}\bigr\Vert \\& \qquad{}+ \biggl\{ \biggl(\rho_{1}\kappa_{1} \biggl(1+ \frac{1}{n} \biggr) \biggr) (\xi_{1}\tau_{1}+ \nu_{1} ) \biggr\} \bigl\Vert x_{2}^{n}-x_{2}^{n-1} \bigr\Vert \biggr]. \end{aligned}$$

Hence,

24 $$ \bigl\Vert x_{1}^{n+1}-x_{1}^{n} \bigr\Vert \leq\theta_{1}^{n}\bigl\Vert x_{1}^{n}-x_{1}^{n-1}\bigr\Vert + \vartheta_{1}^{n}\bigl\Vert x_{2}^{n}-x_{2}^{n-1} \bigr\Vert , $$

where

$$\theta_{1}^{n}=\frac{1}{(\rho_{1} \varrho_{1}-3 \varepsilon_{1})\beta_{1}^{2}} \biggl\{ \kappa_{1}\sigma_{1}+ \biggl(\rho_{1} \kappa_{1} \biggl(1+\frac{1}{n} \biggr) \biggr) (\mu_{1} \delta_{1}+\zeta_{1} )+\rho_{1} \gamma_{1} \biggr\} $$

and

$$\vartheta_{1}^{n}=\frac{1}{(\rho_{1} \varrho_{1}-3 \varepsilon_{1})\beta_{1}^{2}} \biggl\{ \biggl( \rho_{1}\kappa_{1} \biggl(1+\frac{1}{n} \biggr) \biggr) (\xi_{1}\tau _{1}+\nu_{1} ) \biggr\} . $$

Secondly, it follows from (16) of Algorithm 4.1, for all $y_{2} \in K_{2}$, that

$$\begin{aligned}& \rho_{2} N_{2} \bigl(z_{2}^{n}, \eta_{2} \bigl(f_{2}(y_{2}),f_{2} \bigl(x_{2}^{n} \bigr) \bigr) \bigr)+ \bigl\langle g_{2} \bigl(f_{2} \bigl(x_{2}^{n} \bigr) \bigr)-g_{2} \bigl(f_{2} \bigl(x_{2}^{n-1} \bigr) \bigr) + \rho _{2} \bigl\{ M_{2} \bigl(u_{2}^{n-1},v_{2}^{n-1} \bigr) \\& \quad{}+w_{2}^{n-1} \bigr\} ,\eta_{2} \bigl(f_{2}(y_{2}),f_{2} \bigl(x_{2}^{n} \bigr) \bigr) \bigr\rangle +\rho_{2} \bigl\{ \phi_{2} \bigl(x_{1}^{n-1},y_{2} \bigr)-\phi_{2} \bigl(x_{2}^{n-1},x_{2}^{n} \bigr) \\& \quad{}+\alpha_{2} \bigl\Vert f_{2}(y_{2})-f_{2} \bigl(x_{2}^{n} \bigr)\bigr\Vert ^{2} \bigr\} \geq0 \end{aligned}$$

and

$$\begin{aligned}& \rho_{2} N_{2} \bigl(z_{2}^{n+1}, \eta_{2} \bigl(f_{2}(y_{2}),f_{2} \bigl(x_{2}^{n+1} \bigr) \bigr) \bigr)+ \bigl\langle g_{2} \bigl(f_{2} \bigl(x_{2}^{n+1} \bigr) \bigr)-g_{2} \bigl(f_{2} \bigl(x_{2}^{n} \bigr) \bigr) + \rho _{2} \bigl\{ M_{2} \bigl(u_{2}^{n},v_{2}^{n} \bigr) \\& \quad{}+w_{2}^{n} \bigr\} ,\eta_{2} \bigl(f_{2}(y_{2}),f_{2} \bigl(x_{2}^{n+1} \bigr) \bigr) \bigr\rangle +\rho_{2} \bigl\{ \phi_{2} \bigl(x_{2}^{n},y_{2} \bigr)-\phi_{2} \bigl(x_{2}^{n},x_{2}^{n+1} \bigr) \\& \quad{}+\alpha_{2} \bigl\Vert f_{2}(y_{2})-f_{2} \bigl(x_{2}^{n+1} \bigr)\bigr\Vert ^{2} \bigr\} \geq0. \end{aligned}$$

Using the same arguments as above, the imposed conditions on $N_{2}$, $g_{2}$, $\eta_{2}$, $f_{2}$, $A_{2}$, $T_{2}$, $S_{2}$, and Algorithm 4.1, we obtain

25 $$ \bigl\Vert x_{2}^{n+1}-x_{2}^{n} \bigr\Vert \leq\theta_{2}^{n}\bigl\Vert x_{2}^{n}-x_{2}^{n-1}\bigr\Vert + \vartheta_{2}^{n} \bigl\Vert x_{1}^{n}-x_{1}^{n-1} \bigr\Vert , $$

where

$$\theta_{2}^{n}=\frac{1}{(\rho_{2}\varrho_{2}-3 \varepsilon_{2})\beta_{2}^{2}} \biggl\{ \kappa_{2}\sigma_{2}+ \biggl(\rho_{2} \kappa_{2} \biggl(1+\frac{1}{n} \biggr) \biggr) (\xi_{2} \tau_{2}+\nu_{2} )+\rho_{2}\gamma_{2} \biggr\} $$

and

$$\vartheta_{2}^{n}=\frac{1}{(\rho_{2} \varrho_{2}-3 \varepsilon_{2})\beta_{2}^{2}} \biggl\{ \biggl( \rho_{2}\kappa_{2} \biggl(1+\frac{1}{n} \biggr) \biggr) (\mu_{2}\delta _{2}+\zeta_{2} ) \biggr\} . $$

Adding (24) and (25), we have

26 $$\begin{aligned} \bigl\Vert x_{1}^{n+1}-x_{1}^{n} \bigr\Vert +\bigl\Vert x_{2}^{n+1}-x_{2}^{n} \bigr\Vert \leq& \bigl\{ \theta_{1}^{n}+ \vartheta_{2}^{n} \bigr\} \bigl\Vert x_{1}^{n}-x_{1}^{n-1} \bigr\Vert + \bigl\{ \theta_{2}^{n}+\vartheta_{1}^{n} \bigr\} \bigl\Vert x_{2}^{n}-x_{2}^{n-1} \bigr\Vert \\ \leq& \max \bigl\{ \widetilde{\theta}_{1}^{n},\widetilde{\theta}_{2}^{n} \bigr\} \bigl\{ \bigl\Vert x_{1}^{n}-x_{1}^{n-1}\bigr\Vert +\bigl\Vert x_{2}^{n}-x_{2}^{n-1}\bigr\Vert \bigr\} , \end{aligned}$$

where

$$\begin{aligned} \widetilde{\theta}_{1}^{n}= \bigl\{ \theta_{1}^{n}+ \vartheta_{2}^{n} \bigr\} =&\frac {1}{(\rho_{1} \varrho_{1}-3 \varepsilon_{1})\beta_{1}^{2}} \biggl\{ \kappa_{1}\sigma _{1}+ \biggl(\rho_{1} \kappa_{1} \biggl(1+\frac{1}{n} \biggr) \biggr) (\mu _{1}\delta_{1}+\zeta_{1} )+\rho_{1} \gamma_{1} \biggr\} \\ &{}+\frac{1}{(\rho_{2} \varrho_{2}-3 \varepsilon_{2})\beta_{2}^{2}} \biggl\{ \biggl(\rho_{2}\kappa_{2} \biggl(1+\frac{1}{n} \biggr) \biggr) (\mu_{2}\delta _{2}+\zeta_{2} ) \biggr\} \end{aligned}$$

and

$$\begin{aligned} \widetilde{\theta}_{2}^{n}= \bigl\{ \theta_{2}^{n}+ \vartheta_{1}^{n} \bigr\} =&\frac {1}{(\rho_{2}\varrho_{2}-3 \varepsilon_{2})\beta_{2}^{2}} \biggl\{ \kappa_{2}\sigma _{2}+ \biggl(\rho_{2} \kappa_{2} \biggl(1+\frac{1}{n} \biggr) \biggr) (\xi_{2} \tau _{2}+\nu_{2} )+\rho_{2}\gamma_{2} \biggr\} \\ &{}+\frac{1}{(\rho_{1} \varrho_{1}-3 \varepsilon_{1})\beta_{1}^{2}} \biggl\{ \biggl(\rho_{1}\kappa_{1} \biggl(1+\frac{1}{n} \biggr) \biggr) (\xi_{1}\tau_{1}+ \nu _{1} ) \biggr\} . \end{aligned}$$

Letting

$$\begin{aligned}& \widetilde{\theta}_{1}=\frac{1}{(\rho_{1} \varrho_{1}-3 \varepsilon_{1})\beta _{1}^{2}} \bigl\{ \kappa_{1} \sigma_{1}+\rho_{1}\kappa_{1} (\mu_{1} \delta_{1}+\zeta _{1} )+\rho_{1} \gamma_{1} \bigr\} +\frac{1}{(\rho_{2} \varrho_{2}-3 \varepsilon_{2})\beta_{2}^{2}} \bigl\{ \rho_{2} \kappa_{2} (\mu_{2}\delta_{2}+\zeta _{2} ) \bigr\} \end{aligned}$$

and

$$\begin{aligned}& \widetilde{\theta}_{2}=\frac{1}{(\rho_{2}\varrho_{2}-3 \varepsilon_{2})\beta _{2}^{2}} \bigl\{ \kappa_{2} \sigma_{2}+\rho_{2}\kappa_{2} (\xi_{2} \tau_{2}+\nu_{2} )+\rho_{2}\gamma_{2} \bigr\} +\frac{1}{(\rho_{1} \varrho_{1}-3 \varepsilon _{1})\beta_{1}^{2}} \bigl\{ \rho_{1}\kappa_{1} ( \xi_{1}\tau_{1}+\nu_{1} ) \bigr\} , \end{aligned}$$

it can easily be seen that $\widetilde{\theta}_{1}^{n}\rightarrow\widetilde{\theta}_{1}$ and $\widetilde{\theta}_{2}^{n} \rightarrow\widetilde{\theta}_{2}$, as $n\rightarrow\infty$. Taking into account the condition (18), we conclude that $\max \{\widetilde{\theta}_{1},\widetilde{\theta}_{2} \}<1$. Hence, it follows from (26) that $\{(x_{1}^{n},x_{2}^{n})\}$ is a Cauchy sequence in $K_{1}\times K_{2}$; now suppose that $(x_{1}^{n},x_{2}^{n} )\rightarrow (x_{1},x_{2} )\in K_{1}\times K_{2}$, as $n \rightarrow\infty$. By Algorithm 4.1 and $\mathcal {D}$-Lipschitz continuity of $T_{i}, S_{i}, B_{i}$ and $A_{i}$, for each $i \in I$, we have

$$\begin{aligned}& \bigl\Vert u_{i}^{n+1}-u_{i}^{n}\bigr\Vert \leq \biggl(1+\frac{1}{n+1} \biggr)\mathcal{D} \bigl(T_{i} \bigl(x_{1}^{n+1} \bigr),T_{i} \bigl(x_{1}^{n} \bigr) \bigr) \\ & \phantom{\bigl\Vert u_{i}^{n+1}-u_{i}^{n}\bigr\Vert }\leq \biggl(1+\frac{1}{n+1} \biggr)\delta_{i}\bigl\Vert x_{1}^{n+1}-x_{i}^{n}\bigr\Vert ; \\ & \bigl\Vert v_{i}^{n+1}-v_{i}^{n}\bigr\Vert \leq \biggl(1+\frac{1}{n+1} \biggr)\mathcal{D} \bigl(S_{i} \bigl(x_{2}^{n+1} \bigr),S_{i} \bigl(x_{2}^{n} \bigr) \bigr) \\ & \phantom{\bigl\Vert v_{i}^{n+1}-v_{i}^{n}\bigr\Vert }\leq \biggl(1+\frac{1}{n+1} \biggr)\tau_{i}\bigl\Vert x_{2}^{n+1}-x_{2}^{n}\bigr\Vert ; \\ & \bigl\Vert w_{i}^{n+1}-w_{i}^{n}\bigr\Vert \leq \biggl(1+\frac{1}{n+1} \biggr)\mathcal{D} \bigl(B_{i} \bigl(x_{1}^{n+1},x_{2}^{n+1} \bigr),B_{i} \bigl(x_{1}^{n},x_{2}^{n} \bigr) \bigr) \\ & \phantom{\bigl\Vert w_{i}^{n+1}-w_{i}^{n}\bigr\Vert }\leq \biggl(1+\frac{1}{n+1} \biggr) \bigl(\zeta_{i}\bigl\Vert x_{1}^{n+1}-x_{1}^{n}\bigr\Vert +\nu_{i}\bigl\Vert x_{2}^{n+1}-x_{2}^{n} \bigr\Vert \bigr); \end{aligned}$$

and

$$\begin{aligned} \bigl\Vert z_{i}^{n+1}-z_{i}^{n}\bigr\Vert \leq& \biggl(1+\frac{1}{n+1} \biggr)\mathcal{D} \bigl(A_{i} \bigl(x_{i}^{n+1} \bigr),A_{i} \bigl(x_{i}^{n} \bigr) \bigr) \\ \leq& \biggl(1+\frac{1}{n+1} \biggr)\varsigma_{i}\bigl\Vert x_{i}^{n+1}-x_{i}^{n}\bigr\Vert . \end{aligned}$$

Therefore, for each $i \in I$, $\{u_{i}^{n} \}, \{v_{i}^{n} \}, \{w_{i}^{n} \}$, and $\{z_{i}^{n} \}$ are also Cauchy sequences; now assume that $u_{i}^{n} \rightarrow u_{i}$, $v_{i}^{n} \rightarrow v_{i}$, $w_{i}^{n} \rightarrow w_{i}$, and $z_{i}^{n} \rightarrow z_{i}$, as $n \rightarrow\infty$. As $u_{i}^{n} \in T_{i} (x_{1}^{n} )$, we have

$$\begin{aligned} d \bigl(u_{i},T_{i} (x_{1} ) \bigr) =&\bigl\Vert u_{i}-u_{i}^{n}\bigr\Vert +d \bigl(u_{i}^{n},T_{i} \bigl(x_{1}^{n} \bigr) \bigr)+\mathcal{D} \bigl(T_{i} \bigl(x_{1}^{n} \bigr),T_{i} (x_{1} ) \bigr) \\ \leq&\bigl\Vert u_{i}-u_{i}^{n}\bigr\Vert + \delta_{i}\bigl\Vert x_{1}^{n}-x_{1} \bigr\Vert \rightarrow0\quad \mbox{as } n\rightarrow\infty. \end{aligned}$$

Therefore, we deduce that $u_{i} \in T_{i}(x_{1})$. Similarly, we can obtain $v_{i} \in S_{i}(x_{2})$, $w_{i} \in B_{i}(x_{1},x_{2})$, and $z_{i} \in A_{i}(x_{i})$, for each $i \in I$.

By Algorithm 4.1, we have

27 $$\begin{aligned}& \rho_{1} N_{1} \bigl(z_{1}^{n+1}, \eta_{1} \bigl(f_{1}(y_{1}),f_{1} \bigl(x_{1}^{n+1} \bigr) \bigr) \bigr)+ \bigl\langle g_{1} \bigl(f_{1} \bigl(x_{1}^{n+1} \bigr) \bigr)-g_{1} \bigl(f_{1} \bigl(x_{1}^{n} \bigr) \bigr) + \rho _{1} \bigl\{ M_{1} \bigl(u_{1}^{n},v_{1}^{n} \bigr) \\& \quad{}+w_{1}^{n} \bigr\} ,\eta_{1} \bigl(f_{1}(y_{1}),f_{1} \bigl(x_{1}^{n+1} \bigr) \bigr) \bigr\rangle +\rho_{1} \bigl\{ \phi_{1} \bigl(x_{1}^{n},y_{1} \bigr)-\phi_{1} \bigl(x_{1}^{n},x_{1}^{n+1} \bigr) \\& \quad{}+\alpha_{1} \bigl\Vert f_{1}(y_{1})-f_{1} \bigl(x_{1}^{n+1} \bigr)\bigr\Vert ^{2} \bigr\} \geq0,\quad\forall y_{1} \in K_{1}; \end{aligned}$$

and

28 $$\begin{aligned}& \rho_{2} N_{2} \bigl(z_{2}^{n+1}, \eta_{2} \bigl(f_{2}(y_{2}),f_{2} \bigl(x_{2}^{n+1} \bigr) \bigr) \bigr)+ \bigl\langle g_{2} \bigl(f_{2} \bigl(x_{2}^{n+1} \bigr) \bigr)-g_{2} \bigl(f_{2} \bigl(x_{2}^{n} \bigr) \bigr) + \rho _{2} \bigl\{ M_{2} \bigl(u_{2}^{n},v_{2}^{n} \bigr) \\& \quad{}+w_{2}^{n} \bigr\} ,\eta_{2} \bigl(f_{2}(y_{2}),f_{2} \bigl(x_{2}^{n+1} \bigr) \bigr) \bigr\rangle +\rho_{2} \bigl\{ \phi_{2} \bigl(x_{2}^{n},y_{2} \bigr)-\phi_{2} \bigl(x_{2}^{n},x_{2}^{n+1} \bigr) \\& \quad{}+\alpha_{2} \bigl\Vert f_{2}(y_{2})-f_{2} \bigl(x_{2}^{n+1} \bigr)\bigr\Vert ^{2} \bigr\} \geq0,\quad\forall y_{2} \in K_{2}. \end{aligned}$$

By using the continuity of $N_{i}$, $M_{i}$, $g_{i}$, $\phi_{i}$, $f_{i}$, and $\eta_{i}$, for each $i \in I$, and since $u_{i}^{n} \rightarrow u_{i}$, $v_{i}^{n} \rightarrow v_{i}$, $w_{i}^{n} \rightarrow w_{i}$, $z_{i}^{n} \rightarrow z_{i}$, and $x_{i}^{n} \rightarrow x_{i}$ for $n \rightarrow\infty$, from (27) and (28), we have, for $\rho_{i} >0$,

$$\begin{aligned}& N_{1}\bigl(z_{1},\eta_{1}\bigl(f_{1}(y_{1}),f_{1}(x_{1}) \bigr)\bigr)+\bigl\langle M_{1}(u_{1},v_{1})+w_{1}, \eta _{1}\bigl(f_{1}(y_{1}),f_{1}(x_{1}) \bigr)\bigr\rangle \\& \quad{}+\phi_{1}(x_{1},y_{1})-\phi_{1}(x_{1},x_{1})+ \alpha_{1}\bigl\| f_{1}(y_{1})-f_{1}(x_{1}) \bigr\| ^{2}\geq 0,\quad\forall y_{1}\in K_{1}, \end{aligned}$$

and

$$\begin{aligned}& N_{2}\bigl(z_{2},\eta_{2}\bigl(f_{2}(y_{2}),f_{2}(x_{2}) \bigr)\bigr)+\bigl\langle M_{2}(u_{2},v_{2})+w_{2}, \eta _{2}\bigl(f_{2}(y_{2}),f_{2}(x_{2}) \bigr)\bigr\rangle \\& \quad{}+\phi_{2}(x_{2},y_{2})-\phi_{2}(x_{2},x_{2})+ \alpha_{2}\bigl\| f_{2}(y_{2})-f_{2}(x_{2}) \bigr\| ^{2}\geq 0,\quad\forall y_{2}\in K_{2}. \end{aligned}$$

Therefore $(x_{1},x_{2},u_{1},u_{2},v_{1},v_{2},z_{1},z_{2},w_{1},w_{2} )$ is the solution of the perturbed system of generalized multi-valued mixed quasi-equilibrium-like problems (1). This completes the proof. □

## Conclusion

In this article, a perturbed system of generalized multi-valued mixed quasi-equilibrium-like problems and a perturbed system of auxiliary generalized multi-valued mixed quasi-equilibrium-like problems are introduced in Hilbert spaces. For the corresponding auxiliary system, we prove the existence of solutions by using relatively suitable conditions. Further, an iterative algorithm is proposed for solving our system and a strong convergence theorem is proved. It is noted that the solution set of our system is larger than the solution set of the system considered by Qiu *et al.* [19], Ding *et al.* [21], and many others. Also, our results improve and extend many well-known results for different systems existing in the literature.
